# Association patterns of cannabis abuse and dependence with risk of problematic non-substance-related dysregulated and addictive behaviors

**DOI:** 10.1371/journal.pone.0255872

**Published:** 2021-08-10

**Authors:** José C. Perales, Antonio Maldonado, Eva M. López-Quirantes, Francisca López-Torrecillas

**Affiliations:** 1 Department of Experimental Psychology, University of Granada, Granada, Spain; 2 Mind, Brain, and Behavior Research Center (CIMCYC), University of Granada, Granada, Spain; 3 Department of Personality, Assessment and Psychological Treatment, University of Granada, Granada, Spain; Radboud University, NETHERLANDS

## Abstract

Co-occurrence of drug misuse with other dysregulated behaviors is common. This study was aimed at exploring the associations between the risk of presenting a clinically relevant condition involving non-substance-related addictive or dysregulated behaviors (as measured by the MultiCAGE CAD-4 screening), and cannabis abuse/dependence (CAST/SDS) scores, and the role of gender therein. Participants were recruited using stratified probabilistic sampling at the University of Granada. Mann-Whitney’s *U* tests were used to compare male and female students in SDS and CAST scores. Associations between gender and MultiCAGE scores were estimated using the γ ordinal correlation index, and tested with χ^2^. For each MultiCAGE dimension, a Poisson-family mixed-effects model was built with either SDS or CAST as the main input variable, while controlling for nicotine and alcohol dependence, and relevant sociodemographic variables. Incidence rate ratios (IRR) were computed for SDS/CAST effects, and the significance threshold was family-wise Bonferroni-corrected. Gender differences were significant for cannabis dependence/abuse and all MultiCAGE scores for non-substance-related conditions, with males showing higher risk scores for excessive gambling, excessive internet use, excessive video gaming, and hypersexuality, and females presenting higher scores in dysregulated eating and compulsive buying. Cannabis dependence and abuse were significantly associated with a higher risk of problematic video gaming. These associations were mostly driven by males. Importantly, although risk of problematic video gaming was specifically associated with cannabis abuse/dependence, there was only a weak non-significant association between problematic video gaming and alcohol use scores. Risk of alcohol use problems, in turn, was strongly associated with all other non-substance-related problems (problematic gambling, excessive Internet use, dysregulated eating, compulsive buying, and hypersexuality). These differential associations can cast light on the etiological similarities and dissimilarities between problematic substance use and putative addictive behaviors not involving drugs.

## Introduction

### Background

The combined use of multiple substances is common among young people in Europe (European Monitoring Centre for Drugs and Drug Addiction [EMCDDA, 1]. Alcohol, cannabis, and tobacco are the drugs most frequently misused by youngsters aged 18–25, and simultaneous use of several substances significantly predicts other risky behaviors (e.g. [2,3]). Polyconsumption exacerbates problems derived from substance use [4–7], and recent studies show gambling-related problems to be predicted, among several regressors, by being male and by dependence scores for alcohol, tobacco, and marijuana [8–11]. In other words, comorbidity of addictive behaviors comprises both substance and non-substance-related problems.

Except for gambling disorder, however, no consensus exists regarding the conceptualization of putative behavioral addictions as *bona fide* addictive disorders. In the last edition of DSM (DSM5), the category *substance-related and addictive disorders* includes a subcategory for *substance use disorders* and another one for *behavioral addictions*. However, the latter, so far, only includes gambling disorder, yet none of the other putative behavioral addictions proposed in the literature. Gambling disorder is thus currently present in the last versions of both the DSM [12] and the ICD [13] as a behavioral addiction, and so it is gaming disorder in the ICD-11 (but not in the DSM-5). Problematic internet use, compulsive buying/shopping, eating/food addiction, and hypersexuality are not currently classified as addictive disorders in any of the main psychiatric nosologies, in spite of which they are frequently conceptualized as addictive by part of the scientific community and the general public [14–16]. Still, and regardless of whether they qualify or not as addictive disorders, there is little doubt that some types of over-engagement in these activities can have a negative impact on wellbeing, health, and social and occupational functioning. In other words, although addiction is still a disputed term in these domains [15], there is some consensus that some of these behavior patterns can become problematic and thus deserve clinical attention [17–19].

The pertinence of exploring co-occurrence of drug abuse with other addictive/dysregulated behaviors not involving the use of drugs is reinforced by recent evidence. For instance, according to some studies (e.g. [20,21]), women with disordered eating and compulsive buying are more likely to have used and misused substances. And some level of co-occurrence of problematic video gaming and substance use also seems to exist, especially in young males [3,10].

For some authors, there are important neurocognitive similarities between excessive involvement in some of these activities and diagnosable addictive disorders (substance use and gambling disorders) [22–25]. These shared mechanisms–including weakened top-down control and executive mechanisms, altered reward processing and sensitivity, attentional biases and cue reactivity, and emotion dysregulation [15,26]–would justify the interest in exploring patterns of co-occurrence across behavioral domains. In other words, a path to better understand these conditions is to carefully quantify their degree of overlap with well-established addictive disorders. However, to date, research has tracked coincidences more closely than potential divergences between substance and non-substance related problems (and between different putative behavioral addictions). In this regard, previous studies have unveiled a distinctive link of problematic gaming and other sedentary leisure activities with cannabis use [3,10]. This link, in turn, seems to be underpinned by personality and individual differences factors that can be dissociated from the ones responsible for a more general and well-known overlap between addictive behaviors [10]. The corroboration of these associations while controlling for relevant confounders is, however, still pending.

Gender can also play a role in this pattern of associations, and gender differences regarding these problematic behaviors have been reported. For example, binge eating and other eating disorders, as well as compulsive buying/shopping are more prevalent in females, whereas gambling and gaming disorder, excessive internet use, hypersexuality, and substance use disorders are more prevalent in males [27–30]. However, the modulating role of gender in associations between different addictive/dysregulated behaviors remains underexplored. Different patterns of co-occurrence across genders would imply that gender-related traits and risk factors underlie such associations, and, consequently, that etiological paths to such behavioral problems (and interventions to prevent or treat them) should also be gender-informed.

### The present study

Here, we focus on cannabis use problems (as measured by the CAST and SDS instruments [32,33]) and their independent associations with an array of non-substance-related behavioral problems (problematic gambling, excessive Internet and video games use, compulsive buying, dysregulated eating, and hypersexuality), measured with the MultiCAGE CAD-4 instrument [34].

The operational definition of non-substance-related problematic behaviors is thus tightly linked here to the instrument used to measure them. The MultiCAGE tool extends the CAGE screening (for risk of alcohol use disorder in primary care settings) [35] to other putative addictive disorders. In all subscales, items refer to subjective perception of the problem, perception by relevant others, feelings of guilt, and lack of control or abstinence symptoms. Criterion validity has been established for alcohol and substance use subscales. For the other subscales, criterion validity has not been established, and a higher score is interpreted as indicating a higher risk of suffering from a condition requiring clinical attention. In terms of delineation of problematic behaviors, the alcohol, illegal drugs, and gambling subscales measure the risk of suffering from substance use and gambling disorders as defined in main psychiatric classifications. Definitions of problematic Internet use, problematic video gaming, compulsive shopping/buying, and hypersexuality mostly overlap with the definition of behavioral addiction according to the components model. Eating problems are defined in a more heterogeneous manner, as they can equally capture restrictive eating behavior, binging, and purging, i.e. they measure eating problems in a way that cannot be equated to eating/food addiction.

Regarding substance use, cannabis-related problems were singled out for the present study for practical and theoretical reasons. Beyond the evidence that cannabis is addictive and thus cannabis abuse presents many of the characteristics observed in other addictive disorders [36,37], cannabis is the most frequently used illegal drug in western countries [1], and has been previously linked to sedentary leisure activities, and, particularly, to digital media use [3,10,38,39]. Therefore, there are at least two reasons why cannabis-related problems are expected to be linked to non-substance-related dysregulated/addictive behaviors. On the one hand, substance-related problems are known to be located at the externalizing end of the externalized-internalized behavior continuum, along with gambling disorder, hypersexuality, and at least some patterns of dysregulated eating [40–45]. And, on the other, the link between cannabis use and sedentary leisure activities could reveal potential shared reward, motivation, and personality-related mechanisms that could be relevant to understand problematic Internet use and video gaming. As noted earlier, a privileged association between excessive video gaming and cannabis use, and the role of personality dimensions therein, have been previously reported [3,10].

Validated scales were used to assess cannabis abuse (CAST; [46]) and severity of dependence (SDS; [47]), and these were separately used as predictors of risk scores of dysregulated behaviors as measured by the MultiCAGE CAD-4 screening (excessive gambling, excessive video gaming, excessive internet use, compulsive buying, dysregulated eating, and hypersexuality), while controlling for relevant confounders. Importantly, the large sample size allowed to take gender into consideration for analyses, and thus to test differences between male and female participants in all measures of interests, but also for different association patterns between cannabis CAST/SDS and MultiCAGE scores across genders. To our knowledge, this is the first attempt to test these associations using comparable instruments for all potentially dysregulated non-substance-related behaviors, while considering the role of gender. Although, in view of the antecedents reviewed earlier, positive associations are expected, especially for tech-related sedentary behaviors, our hypotheses regarding the specific pattern of elevated risks remain open (and thus analyses exploratory).

In summary, to our knowledge, the associations between cannabis use and a sufficiently wide-ranging array of non-substance-related dysregulated/addictive behaviors have never been explored, either by themselves or in relation to gender. The main aim of this study was thus to explore such associations, using comparable measurements for different problematic behaviors, and the role of gender therein. The results yield both theoretical and practical relevance. On the one hand, a precise characterization of the differential associations between non-substance addictive or dysregulated behaviors and cannabis-related problems (including a potential replication of the association between cannabis-related problems and problematic use of the Internet and video games) can help identify separable etiological mechanisms for conditions that are frequently conceptualized and treated as comparable. On the other hand, and beyond theoretical considerations, given the large social acceptance and high rate of use of cannabis among youngsters, a better depiction of its relationships with other mental health hazards is valuable to understand its potential risks, and to design better prevention tools.

## Method

### Participants

The target population for this study were the college students at the University of Granada, Spain. 856 participants from a population of 47096 were recruited using probabilistic stratified sampling from 23 Degree programs [Psychology, Speech therapy, Tourism, English, History, Literature, Business management, Economics, Biology, Physics, Optics, Teacher training (primary education), Teacher training (early childhood education), Education sciences, Law, Medicine, Pharmacy, Social work, Political sciences, Sociology, Computer science, Civil Engineering, and Telecommunications engineering]. Mean age of the sample was 21.12 years (SD = 7.23). 37.62% (322) reported their gender was male, and the rest, female. No participants reported to have a gender identity other than male or female. Participants voluntarily filled all questionnaires, as well as a form with their sociodemographic information in a pen-and-paper format during a break between lectures. Instructions were provided by the second and fourth authors (both of whom are experienced in psychological assessment, and stayed in the room during the whole session and watched it to ensure participants behaved as instructed). All participants were informed about the aims and procedure, and about the possibility of withdrawing from the study at any time. All participants provided a written informed consent. The study was approved by the Human Research Board of the University of Granada. The instruments described below were applied to all participants.

## Measures

### MultiCAGE CAD-4 [34]

This questionnaire is an extension of the CAGE risk screening for alcohol abuse [48], developed to estimate the risk of putative addictive disorders in several behavioral domains (gambling, buying, alcohol use, illegal drugs use, hypersexuality, internet use, video gaming, and eating behavior). According to the original validation criteria, meeting 0 criteria is interpreted as absence of risk, 1 criterion indicates detectable but low risk of problems, and meeting 2 or more criteria is interpreted as indicating a high or very high risk of suffering a clinically significant condition.

The original tool was validated in primary care settings with members of the general population, that is, with people attending public healthcare centers for any reason, not necessarily related to the problematic behaviors assessed. A version of the scale developed specifically for putative technological addictions (including the 8 items for Internet and video games of the version used in this study) was also validated in the general population, outside the primary care setting, and using a snowball sampling method. The Internet and video games subscales of this version yielded psychometric properties very similar to the ones of the original scale [49].

The MultiCAGE CAD-4 has also yielded good reliability for all the subscales, as well as very similar within-subscale factor loadings for all items. Item interchangeability is strongly suggestive of the existence of a unique continuous construct underlying each of the subscales. Reported Cronbach α values were 0.84, 0.73, 0.88, 0.70, 0.82, 0.79, 0.79, and 0.73, and 0.76, for alcohol, gambling, illegal drugs, eating, Internet, video games, buying, and sex subscales, respectively [34]. To our knowledge, criterion validity has been established only for the substance use scales, not the behavioral scales. A cut-off score of 2 was observed to have 92.4% diagnostic sensitivity for alcohol use disorder, 100% for heroin and cannabis use disorder, and 94.1% for cocaine use disorder [34]. Among the non-substance related scales, Internet and video games related problems have been observed to weakly but significantly correlate with executive functioning, social behavior, and emotional control problems, as well as with general mental health [49]. Only non-substance subscales will be used as variables of interest in the present study.

### Fagerström test for nicotine dependence [50]

This instrument measures the intensity of physical addiction to nicotine, and consists of 6 items evaluating the amount of cigarettes smoked, compulsion, and other signs of dependence to nicotine. 4 of these items have two response options and these are recorded as 0/1, the other 2 items have 4 response options, scored 0–3. Item-by-item scores are summed, and the total score is interpreted as a measure of nicotine dependence (< 4: minimal, 4–7: moderate, and > 7: high). In a validation study, Fagerström scores were significantly correlated (0.33 and 0.42) with plasma cotinine levels. Cotinine was used in this validation study instead of nicotine, as it is relatively insensitive to the immediate effects of smoking and constitutes a more stable measure of chronic intake [51]. The Spanish version used here yielded an acceptable Cronbach α value of 0.66 [52].

### Severity of Cannabis Dependence Scale (SDS; [53])

Severity of cannabis dependence was measured using the Spanish version of the SDS [47]. This questionnaire consists of 5 Likert-type items, each in a 0–3 scale. Total score ranges between 0 and 15. Psychometric properties of both the English and the Spanish version are good [47,54], with a Cronbach α of 0.82, and an ICC coefficient of 0.83 for the Spanish version. A cut-off of 3 is normally used to define moderate cannabis dependence, and 7 for severe dependence [46].

### Cannabis Abuse Screening test (CAST; [55])

The CAST is a screening tool for cannabis abuse in the general population that has also been proven valid and reliable for adolescents and young adults [32,47,56]. It consists of 6 items with 5 response option (from 0: never, to 4: very often), referred to the last 12 months. The total score is computed as the sum of the item-by-item responses. The CAST has been validated in adolescent and adult samples [47,57,58] and shows good reliability (AUC = 0.82). Cut-offs of 3 and 7 are used to detect moderate and severe cannabis addiction.

### Statistical analyses

First, gender differences in dependence and abuse of cannabis and in signs of non-substance addictive/dysregulated behaviors were explored. SDS and CAST scores were treated as continuous non-normally distributed variables, and nonparametric Mann-Whitney’s *U* tests were used to compare male and female participants.

MultiCAGE subscale scores range between 0 and 4, depending on the number of items endorsed. Separate scores were thus obtained for the different behavioral domains under scrutiny. The small range of values precludes using MultiCAGE subscales as continuous variables, so their ordinal nature was retained when testing gender effects. Moreover, given that our sample consisted of college students (not exclusively of patients or high-risk individuals), some subscales yielded very low observation frequencies for high scores. In view of that, and in order to ensure a sufficient number of observations per level, high scores were collapsed in a single level. More precisely, individuals in each subscale were classified as without risk of problems (MultiCAGE = 0), with low but detectable risk (1), and with high risk of problems (2–4). As noted above, the high-risk cutoff has only been validated for substance-related MultiCAGE subscales, not for purely behavioral ones. Labels for the latter have been established by mere analogy and must be interpreted with caution. This, however, does not affect to the ordinal nature of the 0–2 scale resulting after collapsing high values. The association between gender and risk for each potentially problematic behavior was estimated using Goodman and Kruskal’s γ ordinal correlation index. This set of analyses was performed in JASP statistical software [59]. Please note that this analysis in particular could have been affected by a reduction of sensitivity of the scale to differences in the high end, so it is important it is confirmed by further analyses (in which all values were retained, and modelled using a Poisson distribution).

To test the associations of CAST and SDS scores with the several MultiCAGE non-substance scales, two sets of regressions were run. As noted above, MultiCAGE subscale scores are discrete (0–4), with most observations being 0, and very few 3 and 4-score observations. In view of this, the R package *lme4* [60] was used to model MultiCAGE scores as Poisson-distributed (i.e. as a count of discrete items endorsed; no collapsing was required in this case). For each MultiCAGE score, a mixed-effects model was built, with the Degree program the participant was enrolled at as the only random-effects factor, age, gender, Fagerström score, and MultiCAGE alcohol score as fixed-effects covariates, and cannabis dependence/abuse (either SDS or CAST total scores) as main fixed-effects input variable. The effects of covariates, including nicotine dependence (Fagerström score) and risk of alcohol use disorder (MultiCAGE–alcohol, i.e. CAGE) will be estimated and reported, and are included in the models to ensure the effects of cannabis problems/dependence, which are the main predictors of interest, are not explained away by concurrent use of other substances. Although comparing the association patterns of non-substance-related problem with different substance-related problems is not the main aim of the present study, differences in such patterns (and what they could imply for further research) will be mentioned and briefly discussed.

To test the role of gender, initial saturated models also included the interactions of gender with SDS/CAST, Fagerström score, and MultiCAGE alcohol score. Each of these interactions was retained in the final regression model only if substantially contributed to model fit, according to a hierarchical comparison (using the Akaike Information Criterion and a χ^2^ test). For the sake of readability, only statistics for predictors included in the final models will be reported here. Incidence rate ratios (IRR) were computed for SDS/CAST effects, and significance was determined by family-wise Bonferroni-corrected *p* < 0.05 (*p*_corrected_ = 0.05/*x*, with *x* representing the number of contrasts in the hypothesis-relevant family). For gender–MultiCAGE correlations, the number of tests to be performed was 6 (one gender effect per MultiCAGE subscale), and the significance threshold was established at p_corrected_ = 0.0083. For SDS/CAST effects, the number of tests considered for family-wise correction will be those of theoretical interest (CAST and SDS direct effects and their interactions with gender in the best-fitting models). As detailed later, family-wise correction yielded in this case a threshold p_corrected_ = 0.0035.

Data and code for these analyses can be accessed at the Open Science Framework website (https://osf.io/2jqnh/).

## Results

Means (standard errors) for males and females, respectively, were 0.960 (0.069) and 0.376 (0.177) for the CAST scale, and 1.236 (0.155) and 0.603 (0.081) for the SDS scale. However, 283 males and 498 females scored 0 in the SDS, and so did 263/478 males/females in the CAST scale. In view of that, comparisons were made using non-parametric Mann-Whitney’s U tests. In the two scales, males scored higher than females (*U* = 90821.5, *p* = 0.005, and *U* = 93218, *p* < 0.005, for SDS and CAST, respectively).

Results of χ^2^ tests for associations between gender and problem risk scores for MultiCAGE non-substance dysregulated/addictive behaviors are shown in Table 1. After family-wise Bonferroni corrections (6-member family, see statistical analyses section and note in Table 1), males were found to present higher risk of problematic gambling, excessive video gaming, hypersexuality, and excessive Internet use, whereas females presented higher risk of buying and eating-related problems.

**Table 1:** Number of males and females in each of the three ranges (0: No risk, 1: Low risk, 2–4: High risk) of MultiCAGE scales for non-substance potentially dysregulated/addictive behaviors, results of a χ^2^ test on the relationship between gender and MultiCAGE level of risk, and gamma (γ) coefficient of ordinal correlation (positive values stand for a higher incidence of risky behaviors in females).

*MultiCAGE*	Gender	No risk	Low risk	High risk	χ^2^ (2)	*p*	γ
Gambling	M	276	26	20	30	< .001*	-0.598
	F	513	13	8			
Eating	M	231	53	38	29.82	< .001*	0.349
	F	292	104	138			
Internet	M	83	91	148	12.84	0.002*	-0.202
	F	198	143	193			
Video games	M	210	48	64	94.98	< .001*	-0.688
	F	489	25	20			
Buying	M	239	54	29	12.7	0.002*	0.227
	F	346	95	93			
Hypersexuality	M	258	34	30	16.92	< .001*	-0.326
	F	474	43	17			

Note: * significant χ^2^ tests after Bonferroni correction (corrected p = 0.05/3 = 0.0083).

**Fig 1:**
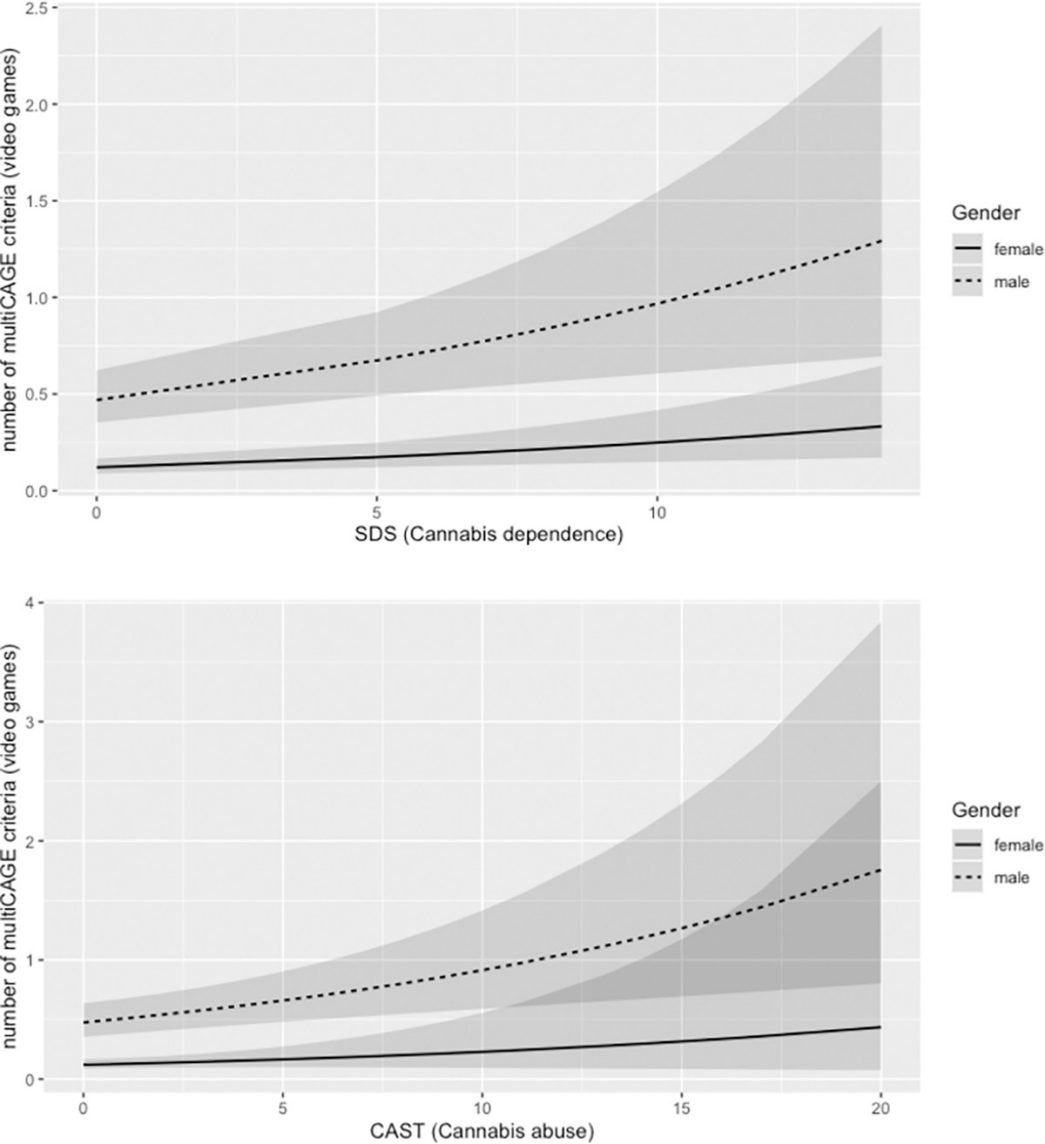
Predicted incidents (number of video games MultiCAGE items endorsed) as a function of cannabis dependence (panel A) and abuse (panel B), and gender.

Results regarding gender effects were confirmed by mixed-effects regressions (Tables 2 and 3). Additionally, cannabis abuse (CAST) and dependence (SDS) were significantly associated with higher risk of problematic video gaming. SDS or CAST interactions with gender did not significantly contributed to model fit, and were left out, except in the case of SDS x Gender on risk of problematic gambling, and CAST x Gender on risk of problematic buying. These effects, however, did not survive the Bonferroni correction. Still, as shown in Fig 1 (model-derived predictions), SDS/CAST significant effects were mostly driven by men. Most likely, the lack of significant interactions resulted from the very infrequent occurrence of females with severe cannabis use and high MultiCAGE scores. All models considered, and their goodness-of-fit indices, as well model comparison tests to reach the best-fitting models included in Tables 2 and 3 are reported in the model comparison file included in the OSF repository for open data and code (https://osf.io/2jqnh/).

**Table 2:** Mixed-effect regressions for non-substance MultiCAGE dysregulated behavior scores over SDS cannabis score and relevant covariates.

	*MC Gambling*		*MC Video gaming*		*MC Internet*		*MC buying*		*MC Eating*		*MC Hypersexuality*	
**Fixed part**	*IRR*	*p*	*IRR*	*p*	*IRR*	*p*	*IRR*	*p*	*IRR*	*p*	*IRR*	*p*
*Intercept*	0.02	<0.001	0.11	<0.001	1.09	0.115	0.38	<0.001	0.71	<0.001	0.09	<0.001
*SDS* (cannabis)	0.98	0.820	1.08	0.002*	1.00	0.997	1.05	0.007	0.96	0.086	1.03	0.215
*Fagerström* (nicotine)	1.26	0.018	0.94	0.302	1.01	0.751	1.18	<0.001	1.03	0.476	1.42	<0.001
*MC Alcohol*	1.97	<0.001	1.15	0.009	1.14	<0.001	1.33	<0.001	1.25	<0.001	1.31	<0.001
*Age*	1.21	0.049	0.91	0.250	0.80	<0.001	0.87	0.020	0.92	0.079	0.93	0.386
*Gender*	5.24	<0.001	3.89	<0.001	1.16	0.028	0.62	<0.001	0.56	<0.001	2.20	<0.001
*SDS x Gender*	1.22	0.018										
*Fagerström x Gender*	0.58	<0.001							1.18	0.016	0.84	0.027
*MC Alcohol x Gender*	0.56	0.002							0.83	0.038		
**Random part**												
σ^2^	2.49		1.59		0.57		1.15		0.91		1.82	
τ_00_	0.32		0.23		0.02		0.11		0.02		0.05	
ICC	0.11		0.13		0.03		0.08		0.02		0.03	
Marginal R^2^/Conditional R^2^	0.234/0.322		0.227/0.324		0.116/0.142		0.150/0.222		0.148/0.167		0.165/0.188	

Note: Significant tests for SDS IRRs after family-wise Bonferroni correction are marked with an asterisk. The tests considered for family-wise correction are those in the grey-shaded area (corrected p = 0.05/14 = 0.0035).

IRR: Incidence Rate Ratio, R^2^: Effect size, Fagerström: Nicotine dependence severity.

**Table 3:** Mixed-effect regressions for non-substance MultiCAGE dysregulated behavior scores over CAST cannabis score and relevant covariates.

	*MC Gambling*		*MC Video gaming*		*MC Internet*		*MC buying*		*MC Eating*		*MC Hypersexuality*	
**Fixed part**	*IRR*	*p*	*IRR*	*p*	*IRR*	*p*	*IRR*	*p*	*IRR*	*p*	*IRR*	*p*
*Intercept*	0.02	<0.001	0.11	<0.001	1.05	0.458	0.39	<0.001	0.71	<0.001	0.10	<0.001
*CAST* (cannabis)	0.94	0.465	1.07	<0.001*	1.00	0.944	0.98	0.499	0.96	0.059	1.02	0.302
*Fagerström* (nicotine)	1.26	0.018	0.96	0.422	1.01	0.697	1.19	<0.001	1.03	0.468	1.29	<0.001
*MC Alcohol*	1.96	<0.001	1.17	0.004	1.20	<0.001	1.37	<0.001	1.25	<0.001	1.31	<0.001
*Age*	1.21	0.049	0.91	0.208	0.80	<0.001	0.87	0.019	0.92	0.091	0.92	0.280
*Gender*	5.72	<0.001	3.90	<0.001	1.29	0.003	0.57	<0.001	0.56	<0.001	1.89	<0.001
*CAST x Gender*	1.19	0.064					1.08	0.027				
*Fagerström x Gender*	0.65	0.004							1.19	0.015		
*MC Alcohol x Gender*	0.58	0.003			0.90	0.047			0.83	0.034		
**Random part**												
σ^2^	2.49		1.59		0.57		1.15		0.91		1.82	
τ_00_	0.32		0.25		0.02		0.10		0.02		0.04	
ICC	0.11		0.14		0.03		0.08		0.02		0.02	
Marginal R^2^/Conditional R^2^	0.232/0.319		0.224/0.330		0.122/0.148		0.161/0.227		0.150/0.168		0.138/0.156	

Note: Significant tests for CAST IRRs after family-wise Bonferroni correction are marked with an asterisk. The tests considered for family-wise correction are those in the grey-shaded area (corrected p = 0.05/14 = 0.0035).

IRR: Incidence Rate Ratio, R^2^: effect size, Fagerström: Nicotine dependence severity.

## Discussion

Addictive and other dysregulated behaviors (including putative behavioral addictions) frequently co-occur. Previous studies have shown substantial associations between alcohol, tobacco, and cannabis misuse, and between these, gambling problems, and other externalizing psychopathologies. In addition, incidence of drug and gambling-related problems may differ by gender. However, to our knowledge, the associations between cannabis use and a sufficiently wide-ranging array of non-substance addictions and other dysregulated behaviors have never been explored. The main aim of this study was to explore such associations and the role of gender therein. Among substance-related problems, we put our focus on cannabis-related ones because of the high levels of social acceptance and use of cannabis among youngsters, and the previously mentioned association of cannabis problems and some sedentary leisure activities, and, especially, potentially problematic video gaming.

To assess cannabis abuse and dependence, two well-validated instruments, SDS and CAST, were used. Risk of problems associated with gambling, dysregulated eating, excessive video gaming, hypersexuality, compulsive buying, and excessive Internet use were assessed with the corresponding subscales of the MultiCAGE CAD-4 screening tool. Given that this questionnaire implements the same assessment method, in the same scale (0 to 4 items endorsed by the participants) for all behavioral domains, results across them are easily comparable. The multiCAGE score for illegal drugs was discarded as it is expected to strongly overlap with SDS and CAST (given that cannabis is by far the most frequently consumed illegal drug in Spain). Observed correlation indices of MultiCAGE for illegal drugs with CAST and SDS were 0.492 and 0.431, respectively. However, as MultiCAGE for illegal drugs is sensitive to potential use of other substances, using it as variable of interest would be hardly interpretable. MultiCAGE alcohol score (i.e. the CAGE screening scale) was used as a control variable, along with nicotine dependence (Fagerström test), age, and the Degree program participants were enrolled at. The effect of gender was considered by itself and in interaction with the other relevant predictors.

The two cannabis measures were strongly intercorrelated (Kendall’s τ = 0.793), but they were also related to nicotine dependence (Kendall’s τ = 0.307 and 0.330 for SDS and CAST, respectively), and alcohol related problems (Kendall’s τ = 0.172 and 0.161 for SDS and CAST, respectively; all correlations are significant). These results align with studies [2,5–7] showing that cannabis use is associated with increases in the severity of abuse of sedative substances (alcohol, benzodiazepines and opioids), and psychostimulants (tobacco, cocaine, and amphetamines). In relation to the aims of the present study, these associations also highlight the need to control for nicotine and alcohol-related problems when independently assessing cannabis-related hazards.

As expected, scores for cannabis abuse and dependence were higher in males than in females. So were gambling, video gaming, hypersexuality, and Internet-related problems (see [20,61,62], for similar results). Eating behavior and buying problems, however, were more prevalent in females than in males (see also [20,21]).

Also in accordance with our previous results with the MultiCAGE scale [63,64], risk of Internet-related problems, in both genders, excessive video gaming, in males, and eating-related problems, in females, had especially high prevalence rates. Please note, however, that this high prevalence, especially for excessive Internet use and video gaming, has been partially attributed to a low threshold for pathology detection [63,65]. In addition, the motives for Internet over-engagement seem to differ between males and females [66–68].

Beyond drug use, cannabis abuse and dependence were similarly indicative of a higher risk of video gaming-related problems. Although alcohol misuse and nicotine dependence were not the main focus of the present work, Tables 2 and 3 show that associations for SDS/CAST are more circumscribed than the ones of alcohol (which is associated to virtually all other signs of dysregulated behavior). SDS/CAST associations are also quite different from the ones of nicotine (which is independently associated only with hypersexuality and compulsive buying). This pattern of links is especially relevant in diagnostic and etiological terms, since gambling disorder and gaming disorder are the only behavioral addictions recognized as such in the DSM5 and ICD11 classifications, respectively (not without much discussion and criticism for the latter [69]).

On the one hand, taken as a whole, the more generalized associations between substance-related problems and putative behavioral addictions reinforce the idea that there are common transdiagnostic factors that cut through externalizing psychopathologies, and account for comorbidities between them [70]. A good candidate to play that role is malfunctioning of emotion regulation mechanisms. More specifically, recent works have proposed affect-driven impulsivity or *urgency* (the proneness to rash action when experiencing strong positive or negative emotions) as a proxy to this type of emotion dysregulation. Indeed, recent theoretical developments attribute a crucial etiological function to affect-driven impulsive action in the vulnerability and emergence of substance use disorders, antisocial/aggressive behavior, and gambling disorder [71,72].

On the other hand, among multiCAGE dimensions, the video gaming score is the one least associated with alcohol-related and other externalizing problems. Yet, it is the only one clearly associated with cannabis abuse and dependence. A similar dissociation has been previously reported [3,10], and strongly suggests diverging etiologies for different patterns of behavior customarily considered as addictive. More specifically, it seems unlikely for the association between cannabis and video gaming-related problems to be rooted in externalization and related traits (at difference with what seems to happen for the comorbidity between alcohol use disorder, gambling disorder, and conduct problems). This warrants further investigation on the potential motivational and reward-related mechanisms that could account for it. Tentatively, the observed overlap between video gaming and cannabis use problems could be related instead to shared individual differences factors as lower persistence and sensation seeking, and higher stress sensitivity [73–75], or motives related to the dissociative/immersive effects of both cannabis and video games [76,77].

## The role of gender

Gender differences were observed in all relevant measures (see Table 1). However, we did not detect significant differences between males and females regarding the strength of relationships between cannabis abuse/dependence and MultiCAGE scores. Whether this absence of significant interactions is genuine, or due to the low joint incidence of comorbid severe drug dependence and other dysregulated/addictive behaviors in female participants, remains an open question. In case these interactions were confirmed by future research, they would support the idea that potentially addictive behaviors might obey to partially different mechanisms in males and females, which could also lead to different clinical implications [27,29,43].

More specifically, females in our study presented higher scores for risk of problematic eating behaviors and compulsive buying, whereas males presented higher scores for risk of cannabis abuse and dependence, and also for excessive gambling, excessive internet use, excessive video gaming and hypersexuality. Explanations for these differences remain a matter of discussion [27–31]. Higher prevalence of addictive behaviors in males seems in line with their general higher risk of externalizing problems, and their higher scores in their related personality traits [78,79]. However, compulsive buying is more prevalent in females, despite the fact it also overlaps with externalizing behaviors and related traits, and evidence shows it comprises at least some of the components considered prototypical of behavioral addictions. These components include weakened control despite negative consequences, tolerance, withdrawal, and craving [80–82]. Its higher prevalence in females has been related to the observation that buying is used as an overt emotional regulation strategy to cope with or scape from negative affective states [28]. In a similar vein, disordered eating-related behaviors have been linked to emotional avoidance and dysfunctional coping strategies [83,84], as well as self-image distortions and social pressures that affect females to larger degree than males. Unfortunately, the imprecise delimitation of eating problems in the MultiCAGE scale (mixing behaviors of different sorts) does not allow us to make any theoretical interpretations beyond these very general similarities with previous research.

Beyond explanatory accounts, however, these differences are crucial for prevention and treatment, as there is little gender-specific health care in this domain [79]. Current policies regarding prevention, management and therapeutic treatment mostly neglect the fact that prevalence rates and risk indices for addiction and self-regulation-related problems could be gender-specific.

Our results also suggest that differential prevalence rates and etiological pathways can give rise to different patterns of associations among problems in different behavioral domains across genders. Neglecting this is probably precluding a more efficient health care approach, as gender-based prevention measures or therapies are bound to be more effective than the usual ‘one-size-fits all’. Addressing gender in health and health care requires new approaches at many levels, from training health personnel to the development of clinical tools.

## Limitations and strengths

This is a cross-sectional study, which means that the causal direction of links cannot be established. It would be premature to assert that cannabis use has an etiological role in behavioral addictions, or the other way around. Alternatively, current evidence seems to support the idea that drug and non-drug-related behavior regulation problems could share transdiagnostic factors that explains the common variance and their overlap of vulnerabilities and symptomatology, as well as their high degree of comorbidity.

Among limitations, we must also note that generalizability beyond the population of reference is not ensured (for example to non-University enrolled persons of the same age). Relatedly, the low frequencies in the high-severity or pathological levels of the different scales may have restricted the range of observations and thus reduced correlations to some degree. Finally, scales measuring putative behavioral addictions based on items developed for scales for substance use disorders (e.g. from CAGE-alcohol to MultiCAGE-video gaming or internet “addictions”) may yield a large number of “false positives” and increase the risk of pathologising normal behavior [21,65].

Nevertheless, this study also has some remarkable strengths. First, assessments were carried out for a large sample of participants, and this sample was selected to be as representative as possible of the population of reference. Second, at difference with most previous studies, we carefully controlled for potential confounders, using an analysis method that allows to control for both quantitative covariates (i.e. socio-demographic variables, levels of misuse of other drugs) and discrete sources of covariance in the data (i.e. degree). And, finally, responses in the MultiCAGE were modeled to follow Poisson distributions (accumulation of occurrences, namely clinical criteria), using a generalized mixed-effects modeling approach, which maximizes the reliability of results.
