# Supplementary material for: Association patterns of cannabis abuse and dependence with risk of problematic non-substance-related dysregulated and addictive behaviors
Source: PLoS One. 2021 Aug 10;16(8):e0255872. doi: 10.1371/journal.pone.0255872 (PMC8354435; doi:10.1371/journal.pone.0255872)
Supplement: Attachment — Submitted filename: Response to Reviewers.docx.doc [file pone.0255872.s001.doc]

**Reviewer 1**

We appreciate the Reviewer’s insightful feedback. All their concerns have been addressed in a point-by-point fashion, as detailed below. Changes made in the manuscript in response to Reviewer 1’s comments are highlighted in yellow in the revised version of the manuscript.

**1) The aims were not clearly stated until the discussion. The introduction would benefit from a clear statement of the study aim.**

*We have rewritten the introduction to clearly establish the aims of the study. Please see the parts highlighted in yellow.*

**2) In the first paragraph of the introduction (page 3) “Across countries, alcohol, cannabis and tobacco are the drugs most likely to be misused by youngsters”. Please specify the age group which is being referred to as ‘youngsters’.**

*Done*

**4) The concepts of disordered eating, compulsive buying, problematic video gaming, hypersexuality, excessive internet use and problem gambling are not defined in the introduction. The article would be clearer if these key variables were defined and an explanation was given for why they are problematic/if they are a recognized disorder in the introduction.**

*Please see the rewritten parts in the introduction, especially the following ones:*

*“With the exception of gambling disorder, however, no consensus exists regarding the conceptualization of putative behavioral addictions as bona fide addictive disorders. Consequently, whereas (a) gambling disorder is present in the last versions of both the DSM [12] and the ICD-11 [13], (b) gaming disorder is included in the ICD, but not in the DSM, and (c) problematic internet use, compulsive buying/shopping, eating/food addiction, and hypersexuality are not currently included in any of the current psychiatric nosologies. In spite of this, these behaviors are frequently conceptualized in the literature as addictive [14-16]. Still, it is more generally accepted that, regardless of whether or not they qualify as mental disorders, these conditions can course with over-engagement, behavioral and cognitive salience, perceived lack of control, unsuccessful attempts to quit or reduce the time spent in the problematic activity, negative emotional states associated with abstinence, social, academic, or occupational problems related to the behavior, and negative impact on the life of the affected person, reflected in impaired health or altered social functioning [17].”*

*“The operational definition of non-substance-related problematic behaviors is thus tightly linked here to the instrument used to measure them. The MultiCAGE tool extends the CAGE screening (for risk of alcohol use disorder in primary care settings) [35] to other putative addictive disorders. In all subscales, items refer to subjective perception of the problem, perception by relevant others, feelings of guilt, and lack of control or abstinence symptoms. Criterion validity (2 or more positive responses) has been established for alcohol and substance use disorders. For the other subscales, a higher score is interpreted as indicating a higher risk of suffering from a condition requiring clinical attention. In terms of delineation of problematic behaviors, the alcohol, illegal drugs, and gambling subscales measure the risk of suffering from substance use and gambling disorders as defined in main psychiatric classifications. Definitions of problematic Internet use, problematic video gaming, compulsive shopping/buying, and hypersexuality mostly overlap with the definition of behavioral addiction according to the components model [34] (taking into account that excessive Internet use is defined in a content-independent way, and hypersexuality includes both in-person and Internet-based, as well as both interactive and solitary sexual activity). Eating problems are defined in a more heterogeneous manner, as they can equally capture restrictive eating behavior, binging, and purging, i.e. they measure eating problems in a way that cannot be equated to eating/food addiction.”*

**5) Gender was a key variable in the study, however the problematic behaviors are not discussed in relation to gender. Placing more focus on this area would improve understanding of why it was considered in this study.**

*A more detailed consideration of gender has been included in the introduction:*

*“Complementarily, it is also important to explore the potential role of gender in these overlaps. Gender differences in these problematic behaviors have been reported. For example, binge eating and other eating disorders, as well as compulsive buying/shopping are more prevalent in females, whereas gambling and gaming disorder, excessive internet use, hypersexuality, and substance use disorders are more prevalent in males [27-30]. However, the modulating role of gender in associations between different addictive/dysregulated behaviors remains underexplored. Different patterns of co-occurrence across genders would imply that gender-related traits and risk factors underlie such associations, and, consequently, that etiological paths to such behavioral problems (and interventions to prevent or treat them) should also be gender-informed.”*

**6) The sentence which reads “Validated scales were used to assess cannabis use……” (pg4) should have a reference to support this claim.**

*The corresponding references have been added.*

**7) It was not clear how the two cannabis scales were given to participants. Did all participants receive both? In which case please explain why two scales were used. Or were participants split into groups where some received the CAST and others received the SDS? If that is the case please provide justification and provide descriptions for the two groups.**

*We have now made explicit that all participants went through all the assessments. We had a number of reasons for keeping both questionnaires in analyses. Firstly, although both are short assessments, CAST measures the risk of cannabis abuse (Legleye et al., 2007), i.e. it is a screening instrument, whereas the SDS assesses cannabis dependence (Martin et al., 2006). Having the output construct operationalized in two different ways certainly increases the probability that results differ across measures but, at the same time, increases the robustness of coincident patterns of results. Additionally, if, once the two tools have been administered, we selected only one for reporting, that would put us in a serious risk of report bias.*

*Legleye, S., Karila, L., Beck, F., & Reynaud, M. (2007). Validation of the CAST, a general population cannabis abuse screening test. Journal of Substance use, 12(4), 233-242. doi: 10.1080/14659890701476532*

*Martin, G., Copeland, J., Gates, P., & Gilmour, S. (2006). The severity of dependence scale (SDS) in an adolescent population of cannabis users: Reliability, validity and diagnostic cut-off. Drug and Alcohol Dependence, 83(1), 90-93. doi:10.1016/j.drugalcdep.2005.10.014*

**8) Please state how many schools were included in the school-by-school analysis, as this is later used in statistical models:**

*As specified (and corrected) in the revised version of the manuscript, we included 23 Degree programs from 10 different schools. The variable used as random-effects factor for analysis was Degree, not school.*

**9) In the discussion of the MultiCAGE CAD-4 (pg 6): Please note that the cut-off score or 2 is only validated for the substance use scales, not the behavioral scales.**

*Done.*

**10) In the second paragraph when discussing that it is mostly used in primary care settings, please note that it is only validated in these settings and clinical populations, then make the case for why it is still appropriate for your sample.**

*As noted in the revised version of the manuscript, the tool has been validated in primary care settings with members of the general population, that is, with people attending centers for any reason, not necessarily related to the problematic behaviors assessed. A version of the scale developed specifically for putative technological addictions (including the 8 items for Internet and video games of the version used in this study) was validated in the general population, outside the primary care setting, and using a snowball sampling method. The Internet and videogames subscales of this version yielded psychometric properties very similar to the ones of the original scale.*

**11) “This is consistent with other assessment tools for addictive disorders in which severity is estimated as the sum of the number of diagnostic criteria met by the individual assessed” (pg6) this is a bit misleading, please highlight that the majority of the scales which are focused on in this study are behavioral, and do not have a diagnostic criteria to map onto.**

*In order to avoid any baseless generalization, that paragraph has been deleted.*

**12) Please expand the description of the Fagerstrom Test for Nicotine Dependence. For example: range, cut-offs, scoring. Additionally please give some examples of the “good psychometric properties”**

*The following information has been included in the manuscript next information:*

*“This instrument measures the intensity of physical addiction to nicotine, and consists of 6 items evaluating the amount of cigarettes smoked, compulsion, and other signs of dependence to nicotine. 4 of these items have two response options and these are recorded as 0/1, the other 2 items have 4 response options, scored 0-3. Item-by-item scores are summed, and the total score is interpreted as a measure of nicotine dependence (< 4: minimal, 4-7: moderate, and > 7: high). In its original validation, Fagerström scores were significantly correlated (0.33 and 0.42) with plasma cotinine levels [51]. The Spanish version used here yielded an acceptable Cronbach α value of 0.66 [52].”*

**13) In the discussion of the CAST, you state that it has been validated in Spanish adolescents. As your sample is older, please make your case for why it is still appropriate for use. Additionally please state the score range for this measure.**

*That sentence has been rewritten as follows (and relevant references have been provided):*

*“The CAST is a screening tool for cannabis abuse in the general population that has also been proven valid and reliable for adolescents and young adults [32, 47, 56]”*

**14) For clarity specify when Multiscale subscales versus full scale scores are being used. The test appeared to imply a single score.**

*The use of total/subscale scores for all variables of interest have been clarified throughout the paper, e.g.:*

*“MultiCAGE subscale scores range between 0 and 4, depending on the number of items endorsed. Separate scores were thus obtained for the different behavioral domains under scrutiny.”*

**15) On pg7 “and with high risk of clinically significant problems”, please define what constitutes ‘clinically significant problems’ for the target behaviors prior to using this terminology.**

*As noted earlier by the reviewer, the cutoff for high risk of being diagnosed with a psychiatric condition has only been validated for substance-related subscales, not for purely behavioral ones. So that paragraph has been toned down as follows:*

*“The small range of values precludes using MultiCAGE subscales as continuous variables, so their ordinal nature was retained when testing gender effects. Moreover, given that our sample was extracted from the general population (not a particularly high-risk one), some subscales yielded very low observation frequencies for high scores. In view of that, and in order to ensure a sufficient number of observations per level, high scores were collapsed in a single level. More precisely, individuals in each subscale were classified as without risk of problems (MultiCAGE = 0), with low but detectable risk (1), and with high risk of problems (2-4). As noted above, the high-risk cutoff has only been validated for substance-related MultiCAGE subscales, not for purely behavioral ones. Labels for the latter have been established by mere analogy and must be interpreted with caution. This, however, does not affect to the ordinal nature of the 0-2 scale resulting after collapsing high values.”*

**16) “As noted above….. very few 3 and 4-score observations” (pg7), this sentence requires a citation.**

*The low frequency of high scores is just an observation from this study. More precisely, the gambling subscale yielded n=8/3 for values 3/4, eating 60/13, Internet 121/51, video games 22/11, shopping 35/13, and sex 9/6.*

**17) Please state the alpha levels used after the Bonferroni corrections either here or in the results.**

*Done:*

*“For gender – MultiCAGE correlations, the number of tests to be performed was 6 (one gender effect per MultiCAGE subscale), and the significance threshold was established at pcorrected = 0.0083. For SDS/CAST effects, the number of tests considered for family-wise correction will be those of theoretical interest (CAST and SDS direct effects and their interactions with gender in the best-fitting models). As detailed later, family-wise correction yielded in this case a threshold pcorrected = 0.0035”*

*Please note we have decided to apply a more stringent criteria in the revised version of the manuscript. This was decided on the basis that familywise corrections for SDS/CAST effects should consider the two input variables of interest (CAST and SDS) simultaneously, as well as their interaction with gender in best-fitting models. This yields 14 tests instead of 6, and thus a corrected threshold p of 0.0035 instead of 0.0083.*

**18) Figure 1 either needed numerical labels on the graph points or the means and Cis needed to be written somewhere in the text (pg8).**

*Numerical values in the main text rendered Figure 1 unnecessary, and it has been removed.*

**19) To improve readability, reduce the use of the word “so”.**

*Done.*

**20) If a result were not significant with a Bonferroni corrected p-value, it were not significant in your study and you do not need to discuss it in the results section (pg9). This could be potentially misleading.**

*We agree there is a risk of overinterpretation, but we have left open the possibility that non-significance of gender x CAST/SDS interactions could be due to the low frequency of high scores for females in some scales rendering the analysis unsensitive to such effects despite the large sample size. Our study does not present evidence such interactions exist (“absence of evidence”), but they cannot be interpreted either as “evidence of absence”, that is, as evidence such interactions do not exist.*

*Alderson, P. (2004). Absence of evidence is not evidence of absence. BMJ: British Medical Journal, 328(7438), 476.*

**21) It is interesting that the regression results differed when the CAST or SDS was used. Please discuss your ideas on the cause of this difference.**

*The differences are actually minimal, especially after establishing a stricter criterion for significance (see response to point 17). In general terms, the qualitative patterns of effects shown by SDS and CAST are nearly identical (actually, the correlation between the two is 0.792, which implies that they are virtually measuring the same construct).*

**22) The discussion would benefit from more citations to support the points being made. Tying these findings to the published literature greatly helps understand the potential contribution of the paper.**

*We have expanded the discussion, especially when considering gender-related findings, and included the corresponding references. Moreover, after a detailed search, a number of new references have been added.*

**23) The aim was clearly stated in the discussion, providing good closure to the article. However, I would like more information on why it is important to investigate the ‘wide-ranging array’ of dysregulated behaviors and substance use at the same time (p9).**

*Please see our response to the previous point. In addition, we have substantially rewritten the introduction to provide a stronger background for the aims of the study.*

**24) “MultiCAGE score for illegal drugs was discarded due to its obvious overlap with use of cannabis” (pg 10). What was the correlation of these scales in your study? Additionally, please remove the word ‘obvious’ from this sentence to improve adherence to academic style.**

*That sentence has been rewritten as follows:*

*“MultiCAGE score for illegal drugs was discarded due to the fact that it is expected to strongly overlap with SDS and CAST, as cannabis is the most frequently consumed illegal drug in Spain.”*

*Additionally, we have added the following footnote:*

*“Observed correlation indices of MultiCAGE for illegal drugs with CAST and SDS were 0.492 and 0.431, respectively. However, as MultiCAGE for illegal drugs is sensitive to potential use of other substances, using it as variable of interest would be hardly interpretable.”*

**25) For clarity, more discussion of why alcohol and nicotine were used as controls (pg10), preferably in the introduction or methodology would be useful.**

*The following paragraph has been included in the statistical analyses section:*

*“The effects of covariates, including nicotine dependence (Fagerström score) and risk of alcohol use disorder (MultiCAGE – alcohol, i.e. CAGE) will be estimated and reported, and are included in the models to ensure the effects of cannabis problems/dependence, which are the main predictors of interest, are not explained away by concurrent use of other substances. Although comparing the association patterns of non-substance-related problem with different substance-related problems is not the main aim of the present study, differences in such patterns (and what they could imply for further research) will be mentioned and briefly discussed.”*

*Please consider this in conjunction with the following paragraph from the discussion:*

*“On the other hand, across multiCAGE dimensions, video gaming-related problems risk is the one overlapping least with alcohol-related and other externalizing problems, and, at the same time is the only one clearly associated with cannabis abuse and dependence. This dissociation, previously reported [73], suggests that different patterns of behavior customarily considered as “addictive” could have diverging etiologies. More specifically, it seems unlikely for the association between cannabis and video gaming-related problems to be rooted in externalization and related traits, which warrants further investigation on the potential motivational and reward-related mechanisms that could account for it. In their own words: “the personality of problematic gamblers seemed more similar to the personality of substance users than to that of problematic computer gamers.”, which seems to hold for users of alcohol and other substances, but not for cannabis users. Tentatively, the observed overlap between video gaming and cannabis use problems could be related instead to shared individual differences factors as lower persistence and sensation seeking, and higher stress sensitivity [74-76], or motives related to the dissociative/immersive effects of both cannabis and video games [77, 78].”*

*In general terms, we are convinced the analyses are much more informative if effects of cannabis-related predictors are computed independently of the ones of other substances.*

***26) At the end of the first paragraph of limitations and strengths provide a citation for criticisms of the scales due to false positives (pg 13).***

*The two following references have been added:*

Kardefelt‐Winther, D., Heeren, A., Schimmenti, A., van Rooij, A., Maurage, P., Carras, M., ... & Billieux, J. (2017). How can we conceptualize behavioural addiction without pathologizing common behaviours?. *Addiction, 112(10)*, 1709-1715.

Maraz, A., Király, O., & Demetrovics, Z. (2015). Commentary on: Are we overpathologizing everyday life? A tenable blueprint for behavioral addiction research: The diagnostic pitfalls of surveys: If you score positive on a test of addiction, you still have a good chance not to be addicted. *Journal of Behavioral Addictions, 4(3)*, 151-154.

**Reviewer 2**

We appreciate the Reviewer’s insightful feedback. All their concerns have been addressed in a point-by-point fashion, as detailed below. Changes made in the manuscript in response to Reviewer 2’s comments are highlighted in blue in the revised version of the manuscript.

**1) Third line of 1st paragraph of Introduction: Please substitute the word “youngsters” with something more informative, e.g., adolescents, or persons in the age range X-Y etc.**

*Done:*

*“Across countries, alcohol, cannabis and tobacco are the drugs most frequently misused by youngsters aged 18-25.”*

**2) Second paragraph of Introduction: Please introduce the fact that “gambling disorder” is in DSM5, as an example of how problematic behaviors can eventually be diagnosed. Also introduce the term “substance use disorders”, and not only “addiction”.**

*Done. Please see the following rewritten part:*

*“With the exception of gambling disorder, however, no consensus exists regarding the conceptualization of putative behavioral addictions as bona fide addictive disorders. Consequently, whereas (a) gambling disorder is present in the last versions of both the DSM [12] and the ICD-11 [13], (b) gaming disorder is included in the ICD, but not in the DSM, and (c) problematic internet use, compulsive buying/shopping, eating/food addiction, and hypersexuality are not currently included in any of the current psychiatric nosologies”*

*As well as the following footnote:*

*“In the last edition of DSM (DSM5), the category substance-related and addictive disorders includes a subcategory for substance use disorders and another one for behavioral addictions. However, the latter, so far, only includes gambling disorder, yet none of the other putative behavioral addictions proposed in the literature.”*

**3) Please simplify or split this sentence in the last paragraph of the Introduction, page 3: “And some level of co-occurrence of problematic video gaming with substance use (although lower than the association between use of different substances between them and with gambling) also seems to exist, especially in young males.”**

*The sentence has been rewritten as follows:*

*“And some level of co-occurrence of problematic video gaming and substance use also seems to exist, especially in young males (although this is sizably lower than the associations of the use of different substances between them and with gambling) [3].”*

**4) Please also simplify the next sentence in page 3: “For some authors [17-20], the interest in exploring these associations would be also justified by the potential psychological and neurobiological similarities between excessive involvement in these behaviors and well-established addictions.”**

*That part has been rewritten as follows (please see also our response to Reviewer 3’s comment 1):*

*“For some authors [22-25], there are important neurocognitive similarities between excessive involvement in some of these non-substance-related behaviors and diagnosable addictive disorders (substance use and gambling disorders). These similarities would involve, for instance, top-down control and executive mechanisms, reward processing and sensitivity, attentional biases and cue reactivity, and emotion regulation [15, 26], and would justify the interest in exploring patterns of co-occurrence. In other words, a path to better understand these conditions and, ultimately, to provide evidence regarding their addictive/non-addictive nature, is to carefully quantify their degree of overlap with well-established addictive disorders.”*

**5) “Participants” section in the Methods: Please mention if the questionnaires were administered by an assistant or if they were filled as pen-and paper by the participants themselves. Please explain if the level of privacy during these data collections.**

*The required information has been included in the Methods section.*

**6) First paragraph of page 4: When the authors mention “cannabis abuse and dependence”, are these meant to describe the DSM-IV diagnoses? Please state this clearly in text. Also, in the last sentence in the same paragraph, please briefly mention that the DSM5 and ICD “cannabis use disorder: is also now one diagnosed disorder, with increasing severity based on criteria. This is also relevant to the first sentence of the “Statistical Analysis” section in the Methods (please mention directly what scores were examined).**

*That paragraph has been rewritten as follows:*

*“CAST and SDS are validated scales, that have been successfully tested against DSM5 criteria for cannabis use disorder, and DSM-IV criteria for cannabis dependence, respectively. [32, 33]. The risk of presenting clinically relevant problems involving non-substance-related excessive or dysregulated behaviors will be measured with a single instrument (MultiCAGE CAD-4) [34], which uses the same number of highly similar items for all behavioral domains under scrutiny, and allows to express associations with cannabis abuse/dependence in a common scale, and thus directly compare them.”*

**7) Methods: Could the authors provide a summary Table that shows the main instruments and their basic properties? For example, what they measure, score range, any cutoff values. This may be a helpful tool for the readers. Please also define any Abbreviations used (e.g., “MC” in the Table 2 and 3 legends).**

*We think a table would unnecessarily lengthen the article. However, we have expanded and unified the descriptions of the scales and detailed their psychometric properties.*

**8) Figure 1: Please provide further y-axis tick marks for the variables, and also summarize the statistical analysis of between-gender effects.**

*Figure 1 has been removed and replaced by numerical values in the main text.*

*“Means (standard errors) for males and females, respectively, were 0.960 (0.069) and 0.376 (0.177) for the CAST scale, and 1.236 (0.155) and 0.603 (0.081) for the SDS scale.”*

**9) Discussion, page 11: Please simplify the following sentence, or split into different sentences: “A good candidate to play that role is weakened emotion regulation, and, more specifically, its manifestation as affect-driven impulsivity, namely the proneness to rash action when experiencing strong positive or negative emotions (positive and negative urgency).”**

That sentence has been rewritten as follows:

*“A good candidate to play that role is malfunctioning of emotion regulation mechanisms. More specifically, recent works have proposed affect-driven impulsivity or urgency (the proneness to rash action when experiencing strong positive or negative emotions) as a proxy to this type of emotion dysregulation.”*

**10) Page 12, first sentence in the section on “Gender”: Please change “we were unable
to detect” into “we did not detect”.**

*Done.*

**11) Please mention in the “Limitations” section if the fact that these were enrolled University Students could limit the generalization to non-University enrolled persons of the same age. For example, could non-University enrolled persons have more severe cannabis use disorders? (see a possible suggestion below).**

*Suggestions regarding alternative wordings have been accepted.*

Reviewer 3

We appreciate the Reviewer’s insightful feedback. All their concerns have been addressed in a point-by-point fashion, as detailed below. Changes made in the manuscript in response to Reviewer 2’s comments are highlighted in green in the revised version of the manuscript.

**1) At the end of paragraph 3, the authors comment about interests exploring psychological and neurobiological similarities between co-occurring addictive behaviors. I would like to see the authors expand a bit more on the potential variables that might account for these relations.**

*That paragraph has been rewritten as follows:*

*“For some authors [22-25], there are important neurocognitive similarities between excessive involvement in some of these non-substance-related behaviors and diagnosable addictive disorders (substance use and gambling disorders). These similarities would involve, for instance, top-down control and executive mechanisms, reward processing and sensitivity, attentional biases and cue reactivity, and emotion regulation [15, 26], and would justify the interest in exploring patterns of co-occurrence. In other words, a path to better understand these conditions and, ultimately, to provide evidence regarding their addictive/non-addictive nature, is to carefully quantify their degree of overlap with well-established addictive disorders.”*

**2) How was the Fagerstrom Test for Nicotine Dependence scored/summed?**

*The Fagerstrom scoring system is now described as follows:*

*“This instrument measures the intensity of physical addiction to nicotine, and consists of 6 items evaluating the amount of cigarettes smoked, compulsion, and other signs of dependence to nicotine. 4 of these items have two response options and these are recorded as 0/1, the other 2 items have 4 response options, scored 0-3. Item-by-item scores are summed, and the total score is interpreted as a measure of nicotine dependence (< 4: minimal, 4-7: moderate, and > 7: high).”*

**3) I’m not sure I follow the scoring for the CAST. The authors describe the response scale as 1 to 5, with 1 indicating never. A cut-off score of 3 would indicate then that 3 “never” responses would indicate moderate cannabis addiction. Further explanation is necessary.**

*Thanks for spotting this error. Items are actually scored in a 0-4 scale. This has been corrected.*

**4) What are the limitations of grouping individuals with scores of 2-4 on the Multicage CAD-4 in one group for analyses? While I understand the necessity to ensure adequate observations per group, categorizing potentially dissimilar levels of risk as the same might be problematic. If previous work supports this grouping, it should be cited in this paper.**

Please see our response to comment 15 by reviewer 1. The limitation mentioned by reviewer 3 has been acknowledged in the revised version of the paper.

*“More precisely, individuals in each subscale were classified as without risk of problems (MultiCAGE = 0), with low but detectable risk (1), and with high risk of problems (2-4). As noted above, the high-risk cutoff has only been validated for substance-related MultiCAGE subscales, not for purely behavioral ones. Labels for the latter have been established by mere analogy and must be interpreted with caution. This, however, does not affect to the ordinal nature of the 0-2 scale resulting after collapsing high values. Actually, the association between gender and risk for each potentially problematic behavior was estimated using Goodman and Kruskal’s γ ordinal correlation index. This set of analyses was performed in JASP statistical software [59]. Please note that this analysis in particular could have been affected by a reduction of sensitivity of the scale to differences in the high end, so it is important it is confirmed by further analyses (in which all values were retained, and modelled using a Poisson distribution).”*

**5) It would be helpful for the authors to provide the actual mean scores (either in the text or in the figure) for the CAST and SDS scores by gender, as they are a bit difficult to determine in the figure.**

*The figure has been replaced by numeric values in the main text.*

**6) Additionally, if I am understanding correctly, nearly no one in the sample would qualify as meeting even moderate cannabis abuse/dependence. If this is the case, the discussion might benefit from tempering of some language. For instance, “Cannabis abuse and dependence were more prevalent in males than females.” While it may be true that males outscored females on these measures, would it truly be the case that “abuse and dependence” were more prevalent or that males endorsed more symptoms of problematic cannabis use?**

*6.776% of participants in our sample scored 3 or more in the CAST (i.e. would be classified as suffering from moderate or high cannabis dependence). Still, we agree that sentence should be reworded as follows:*

*“As expected, scores for cannabis abuse and dependence were higher in males than in females.”*

**7) The discussion could benefit from increased explanation for the greater association between cannabis and video gaming compared to alcohol and other externalizing problems. Is there any literature to support why this might be?**

*The discussion has been expanded as follows:*

*“This dissociation, previously reported [73], suggests that different patterns of behavior customarily considered as “addictive” could have diverging etiologies. More specifically, it seems unlikely for the association between cannabis and video gaming-related problems to be rooted in externalization and related traits, which warrants further investigation on the potential motivational and reward-related mechanisms that could account for it. In their own words: “the personality of problematic gamblers seemed more similar to the personality of substance users than to that of problematic computer gamers.”, which seems to hold for users of alcohol and other substances, but not for cannabis users. Tentatively, the observed overlap between video gaming and cannabis use problems could be related instead to shared individual differences factors as lower persistence and sensation seeking, and higher stress sensitivity [74-76], or motives related to the dissociative/immersive effects of both cannabis and video games [77, 78].”*

**8) Within the discussion of the potential transdiagnostic mechanism underlying addictions, are there any gender differences in emotion regulation/impulsivity that might also contribute to this study’s findings?**

*Please see our response to Reviewer 1’s comment 5, and the following rewritten paragraph of the discussion:*

*“More specifically, females in our study presented higher scores for risk of problematic eating behaviors and compulsive buying, whereas males presented higher scores for risk of cannabis abuse and dependence, and also for excessive gambling, excessive internet use, excessive video gaming and hypersexuality. Explanations for these differences remain a matter of discussion [27-31]. Higher prevalence of addictive behaviors in males seems in line with their general higher risk of externalizing problems, and their higher scores in their related personality traits [79]. However, compulsive buying is more prevalent in females, in spite of the fact it also overlaps with externalizing behaviors and related traits, and evidence shows it comprises at least some of the components considered prototypical of behavioral addictions. These components include weakened control despite negative consequences, tolerance, withdrawal, and craving [80-82]. Its higher prevalence in females has been related to the observation that buying is used as an overt emotional regulation strategy to cope with or scape from negative affective states [28]. In a similar vein, disordered eating-related behaviors have been linked to emotional avoidance and dysfunctional coping strategies [83, 84], as well as self-image distortions and social pressures that affect females to larger degree than males. Unfortunately, the imprecise delimitation of eating problems in the MultiCAGE scale (mixing behaviors of quite different sorts) do not allow us to make any theoretical interpretations beyond these general coincidences with previous research.”*

**9) In second paragraph about the MultiCAGE CAD-4, third sentence “additive disorders” should be “addictive.”**

*Corrected.*

**10) In the fourth paragraph of the discussion, last sentence, the authors write, “In addition, the motives for Internet engagement seem to differ between males and females [52].” This sentence could benefit from elaboration on how those motives differ.**

As we are not specifically studying motives in the present work, we have preferred not to lengthen the discussion any further. We have, however, provided some relevant references on the issue.

**11) In the first paragraph of “The role of gender” section, the authors write, “women in our study presented higher scores for eating disorders.” From the MultiCAGE CAD-4 description, it seems that this measure assesses risk of problematic eating behaviors and not eating disorders specifically. If this is the case, this language should be edited.**

*Corrected.*
