# Supplementary material for: Association patterns of cannabis abuse and dependence with risk of problematic non-substance-related dysregulated and addictive behaviors
Source: PLoS One. 2021 Aug 10;16(8):e0255872. doi: 10.1371/journal.pone.0255872 (PMC8354435; doi:10.1371/journal.pone.0255872)
Supplement: Attachment — Submitted filename: Response to reviewers PONE-D-20-32444.R2.doc [file pone.0255872.s002.doc]

**Response to reviewers**

**Reviewer 1**

**1) Overall the paper's readability and clarity needs improvement. We encourage the authors to re-read and review, particularly for excessively long sentences and any colloquial language.**

We have substantially rewritten parts of the introduction and discussion sections to improve readability and flow. Changes are highlighted in yellow.

**2) The first section of the introduction is much improved, however the paper would flow better if this section ended with a short sentence explaining why cannabis use was chosen as the main focus of the study.**

We have added the following lines to the second-to-last paragraph of the background subsection:

*“However, to date, research has tracked coincidences more closely than potential divergences between substance and non-substance related problems (and between different putative behavioral addictions). In this regard, previous studies have unveiled a distinctive link of problematic gaming and other sedentary leisure activities with cannabis use [3, 10]. This link, in turn, seems to be underpinned by personality and individual differences factors that can be dissociated from the ones responsible for a more general and well-known overlap between addictive behaviors [10]. The corroboration of this pattern of associations while controlling for relevant confounders is, however, still pending.”*

**3) The 'Present Study' section could be significantly shortened. Detailed information about the measures used and their psychometrics would be better placed in the methods section, as having this much information in the introduction is distracting and confusing. Additionally, remove the details about the sample for the same reason.**

We have removed the information we have considered non-essential for the arguments made in the introduction.

**4) Page 6 "Actually, a privileged association…." Remove the wordactually. This language is too colloquial for academic writing.**

Done.

**5) Page 8 "Mean age of the sample was 21.12 years (DT= 7.23)" The letters DT appear to be erroneous, please correct.**

Done.

**6) Page 8 rather than "non-assigned gender" use more neutral language such as: have a gender identity other than exclusively male or female.**

Done.

**7) Page 8 in the participants paragraph 'patients' and 'participants' are used interchangeably. Please change all to participants.**

Done.

**8) In the 'Measures' section we appreciate that the authors have gone to effort to include more psychometrics. However, reliability and validity are different concepts. For example for the multiCAGE CAD-4 the authors state that the measure has 'good reliability and validity' then only present internal reliability statistics. Please address both reliability and validity for each measure, with statistical values.**

We have added the required information, when available. See, for example, the rewritten part in the description of MultiCAGE:

*“To our knowledge, criterion validity has been established only for the substance use scales, not the behavioral scales. A cut-off score of 2 was observed to have 92.4% diagnostic sensitivity for alcohol use disorder, 100% for heroin and cannabis use disorder, and 94.1% for cocaine use disorder [34]. Among the non-substance related scales, Internet and video games related problems have been observed to weakly but significantly correlate with executive functioning, social behavior, and emotional control problems, as well as with general mental health [49].”*

**9) Page 9 plasma cotinine levels correlations are used as a validity measure. It is my understanding that cotinine levels only indicate if someone has smoked recently (within a day or so) and is not useful for differentiating addiction. Please, briefly, note why this is a good measure for validation.**

The following sentence has been added to the corresponding section:

*“Cotinine was used in this validation study instead of nicotine, as it is relatively insensitive to the immediate effects of smoking and constitutes a more stable measure of chronic intake [51].”*

(Sentence extracted from the original reference).

**10) Page 10: the sentence "Psychometric analyses……. Cannabis users" needs a citation at the end.**

That sentence has been removed in the revised version.

**11) Page 10: "given that our sample was extracted from the general population" the sample was a college sample not the general population, please clarify.**

Corrected.

**12) In the statistical analysis section, it is unclear why the high scores were collapsed into a single level. The authors describe this as needed to ensure a sufficient number of observations per level, however if this results in the result being sorted into an invalid manner then it calls the interpretability of the results into question. Please justify this decision more fully and acknowledged the lack of pathology in the sample**

**as a limitation of the study.**

The following clarifications have been made in the corresponding section:

*“Please note that this analysis in particular could have been affected by a reduction of sensitivity of the scale to differences in the high end, so it is important it is confirmed by further analyses (in which all values were retained, and modelled using a Poisson distribution).”*

In addition, a mention to the issue has been added to the limitations section.

**13) Overall, the discussion is much improved, however as with the introduction readability could be further improved.**

We have rewritten parts of this section in an attempt to improve flow and readability.

**14) Across page 15/16 there is a sentence containing a quote from another paper speculating the personality of gamblers, please remove the quote, reference the paper and shorten this section to improve clarity.**

That part has been rewritten as follows (the quote has been removed):

*“On the other hand, among multiCAGE dimensions, the video gaming score is the one least associated with alcohol-related and other externalizing problems. Yet, it is the only one clearly associated with cannabis abuse and dependence. A similar dissociation has been previously reported [3, 10], and strongly suggests diverging etiologies for different patterns of behavior customarily considered as addictive. More specifically, it seems unlikely for the association between cannabis and video gaming-related problems to be rooted in externalization and related traits (at difference with what seems to happen for the comorbidity between alcohol use disorder, gambling disorder, and conduct problems).”*

**15) Page 16: the first sentence in "The role of gender" is unclear, please re-write and replace 'intensity' with strength.**

The first two sentences have been rewritten as follows:

*“Gender differences were observed in all relevant measures (see Table 1). However, we did not detect significant differences between males and females regarding the strength of relationships between cannabis abuse/dependence and MultiCAGE scores.”*

**16) "… theoretical interpretations beyond these general coincidences with previous research" change coincidences to similarities.”**

Corrected.

**Reviewer 2**

**1) Discussion, fourth line: "In addition, incidence of drug and gambling-related problems are sensitive to gender". The authors could clarify this sentence, for example: "In addition, incidence of drug and gambling-related problems may differ by gender".**

We have followed the reviewer’s advice at rewriting that sentence.

**Reviewer 3**

**1) The authors have adequately addressed all of my comments and concerns. I have no further comments at this time.**

Many thanks indeed for endorsing our article and for your constructive comments in the previous rounds.
