# Supplementary material for: Association patterns of cannabis abuse and dependence with risk of problematic non-substance-related dysregulated and addictive behaviors
Source: PLoS One. 2021 Aug 10;16(8):e0255872. doi: 10.1371/journal.pone.0255872 (PMC8354435; doi:10.1371/journal.pone.0255872)
Supplement: Peer review history

# Peer review: Decision Letter 0

**DOI:** 10.1371/journal.pone.0255872.r001

**Authors:** Robert Didden

30 Dec 2020

PONE-D-20-32444

Association patterns of cannabis abuse and dependence with risk of problematic non-substancerelated dysregulated and addictive behaviors

PLOS ONE

Dear Dr. López-Torrecillas,

Thank you for submitting your manuscript to PLOS ONE. I have received reviews from three experts in this area. All three reviewers note that the paper addresses an innovative topic and would add to the literature. However, all reviewers see room for improvement, and I agree with them. Therefore, while I cannot accept the current version for publication in Plos One, I would like to invite you to revise the paper on the basis of the comments from the reviewers. There are many comments but I expect that you would have no problems addressing each of them. The reviewers suggest clarification and specification in all sections of the paper and adding information where necessary. 

Please submit your revised manuscript by Feb 13 2021 11:59PM. If you will need more time than this to complete your revisions, please reply to this message or contact the journal office at plosone@plos.org. When you're ready to submit your revision, log on to https://www.editorialmanager.com/pone/ and select the 'Submissions Needing Revision' folder to locate your manuscript file.

Please include the following items when submitting your revised manuscript:

- A rebuttal letter that responds to each point raised by the academic editor and reviewer(s). You should upload this letter as a separate file labeled 'Response to Reviewers'.
- A marked-up copy of your manuscript that highlights changes made to the original version. You should upload this as a separate file labeled 'Revised Manuscript with Track Changes'.
- An unmarked version of your revised paper without tracked changes. You should upload this as a separate file labeled 'Manuscript'.

If you would like to make changes to your financial disclosure, please include your updated statement in your cover letter. Guidelines for resubmitting your figure files are available below the reviewer comments at the end of this letter.

If applicable, we recommend that you deposit your laboratory protocols in protocols.io to enhance the reproducibility of your results. Protocols.io assigns your protocol its own identifier (DOI) so that it can be cited independently in the future. For instructions see: http://journals.plos.org/plosone/s/submission-guidelines#loc-laboratory-protocols

We look forward to receiving your revised manuscript.

Kind regards,

Robert Didden

Academic Editor

PLOS ONE

Journal Requirements:

When submitting your revision, we need you to address these additional requirements.

1. Please ensure that your manuscript meets PLOS ONE's style requirements, including those for file naming. The PLOS ONE style templates can be found at

https://journals.plos.org/plosone/s/file?id=wjVg/PLOSOne_formatting_sample_main_body.pdf and

https://journals.plos.org/plosone/s/file?id=ba62/PLOSOne_formatting_sample_title_authors_affiliations.pdf

2.  In your Data Availability statement, you have not specified where the minimal data set underlying the results described in your manuscript can be found. PLOS defines a study's minimal data set as the underlying data used to reach the conclusions drawn in the manuscript and any additional data required to replicate the reported study findings in their entirety. All PLOS journals require that the minimal data set be made fully available. For more information about our data policy, please see http://journals.plos.org/plosone/s/data-availability.

Upon re-submitting your revised manuscript, please upload your study’s minimal underlying data set as either Supporting Information files or to a stable, public repository and include the relevant URLs, DOIs, or accession numbers within your revised cover letter. For a list of acceptable repositories, please see http://journals.plos.org/plosone/s/data-availability#loc-recommended-repositories. Any potentially identifying patient information must be fully anonymized.

Important: If there are ethical or legal restrictions to sharing your data publicly, please explain these restrictions in detail. Please see our guidelines for more information on what we consider unacceptable restrictions to publicly sharing data: http://journals.plos.org/plosone/s/data-availability#loc-unacceptable-data-access-restrictions. Note that it is not acceptable for the authors to be the sole named individuals responsible for ensuring data access.

We will update your Data Availability statement to reflect the information you provide in your cover letter.

3. Please include captions for your Supporting Information files at the end of your manuscript, and update any in-text citations to match accordingly. Please see our Supporting Information guidelines for more information: http://journals.plos.org/plosone/s/supporting-information.

[Note: HTML markup is below. Please do not edit.]

Reviewers' comments:

Reviewer's Responses to Questions

**Comments to the Author**

1. Is the manuscript technically sound, and do the data support the conclusions?

The manuscript must describe a technically sound piece of scientific research with data that supports the conclusions. Experiments must have been conducted rigorously, with appropriate controls, replication, and sample sizes. The conclusions must be drawn appropriately based on the data presented. 

Reviewer #1: Yes

Reviewer #2: Yes

Reviewer #3: Partly

**********

2. Has the statistical analysis been performed appropriately and rigorously? 

Reviewer #1: Yes

Reviewer #2: Yes

Reviewer #3: Yes

**********

3. Have the authors made all data underlying the findings in their manuscript fully available?

The PLOS Data policy requires authors to make all data underlying the findings described in their manuscript fully available without restriction, with rare exception (please refer to the Data Availability Statement in the manuscript PDF file). The data should be provided as part of the manuscript or its supporting information, or deposited to a public repository. For example, in addition to summary statistics, the data points behind means, medians and variance measures should be available. If there are restrictions on publicly sharing data—e.g. participant privacy or use of data from a third party—those must be specified.

Reviewer #1: Yes

Reviewer #2: Yes

Reviewer #3: Yes

**********

4. Is the manuscript presented in an intelligible fashion and written in standard English?

PLOS ONE does not copyedit accepted manuscripts, so the language in submitted articles must be clear, correct, and unambiguous. Any typographical or grammatical errors should be corrected at revision, so please note any specific errors here.

Reviewer #1: Yes

Reviewer #2: Yes

Reviewer #3: Yes

**********

5. Review Comments to the Author

Please use the space provided to explain your answers to the questions above. You may also include additional comments for the author, including concerns about dual publication, research ethics, or publication ethics. (Please upload your review as an attachment if it exceeds 20,000 characters)

Reviewer #1: Summary:

The current paper examined relations between cannabis abuse, gender and potentially addictive behaviors in a sample of Spanish college students. The students were given the CAST, SDS, the Fagerstrom nicotine dependence scale and the MultiCAGE CAD-4, a measurement of substance and non-substance addictive behaviors. The MultiCAGE CAD-4 subscales used in this study included: gambling, hypersexuality, compulsive shopping, dysregulated eating, problematic video game use and problematic internet use. Chi squares and mixed effects regression models were used to explore these associations. Men were found to be higher risk for problematic gambling, video-gaming, hypersexuality and internet use, while women were higher risk for problematic shopping and eating. Cannabis use on both cannabis measures was significantly associated with problem video gaming, and on the SDS scale cannabis also predicted problematic shopping. This is believed to be the first study to examine cannabis use co-occurrence with such a wide variety of behavioral issues, contributing to the literature in this area. Additionally, the gender findings were described as useful when considering addiction treatment.

Strengths:

The exploration of the co-occurrence of cannabis use and multiple potentially addictive behavioral issues is novel and potentially informative. By examining video-gaming, dysregulated eating, compulsive shopping, problematic internet use and hypersexuality simultaneously with cannabis use the researchers effectively build on, and add to the literature around substance use and behavioral addictions. This study used a Spanish language measure, which itself a meaningful contribution to the majority English language research into addictions. The statistical procedures are appropriate and well explained in the article.

Areas for Improvement:

While the article has empirical value and contributes to the literature, the article would benefit from greater clarity and specificity, especially in the introduction.

Introduction:

o The aims were not clearly stated until the discussion. The introduction would benefit from a clear statement of the study aim.

o In the first paragraph of the introduction (page 3) “Across countries, alcohol, cannabis and tobacco are the drugs most likely to be misused by youngsters”. Please specify the age group which is being referred to as ‘youngsters’.

o The concepts of disordered eating, compulsive buying, problematic video gaming, hypersexuality, excessive internet use and problem gambling are not defined in the introduction. The article would be clearer if these key variables were defined and an explanation was given for why they are problematic/if they are a recognized disorder in the introduction.

o Gender was a key variable in the study, however the problematic behaviors are not discussed in relation to gender. Placing more focus on this area would improve understanding of why it was considered in this study.

o The sentence which reads “Validated scales were used to assess cannabis use……” (pg4) should have a reference to support this claim

Methodology:

o It was not clear how the two cannabis scales were given to participants. Did all participants receive both? In which case please explain why two scales were used. Or were participants split into groups where some received the CAST and others received the SDS? If that is the case please provide justification and provide descriptions for the two groups.

o Please state how many schools were included in the school-by-school analysis, as this is later used in statistical models (p5)

o In the discussion of the MultiCAGE CAD-4 (pg 6):

� Please note that the cut-off score or 2 is only validated for the substance use scales, not the behavioral scales

� In the second paragraph when discussing that it is mostly used in primary care settings, please note that it is only validated in these settings and clinical populations, then make the case for why it is still appropriate for your sample

o “This is consistent with other assessment tools for addictive disorders in which severity is estimated as the sum of the number of diagnostic criteria met by the individual assessed” (pg6) this is a bit misleading, please highlight that the majority of the scales which are focused on in this study are behavioral, and do not have a diagnostic criteria to map onto.

o Please expand the description of the Fagerstrom Test for Nicotine Dependence. For example: range, cut-offs, scoring. Additionally please give some examples of the “good psychometric properties”

o In the discussion of the CAST, you state that it has been validated in Spanish adolescents. As your sample is older, please make your case for why it is still appropriate for use. Additionally please state the score range for this measure.

Statistical analysis:

o For clarity specify when Multiscale subscales versus full scale scores are being used. The test appeared to imply a single score.

o On pg7 “and with high risk of clinically significant problems”, please define what constitutes ‘clinically significant problems’ for the target behaviors prior to using this terminology.

o “As noted above….. very few 3 and 4-score observations” (pg7), this sentence requires a citation.

o Please state the alpha levels used after the Bonferroni corrections either here or in the results.

Results:

o Figure 1 either needed numerical labels on the graph points or the means and Cis needed to be written somewhere in the text (pg8)

o To improve readability, reduce the use of the word “so”

o If a result were not significant with a Bonferroni corrected p-value, it were not significant in your study and you do not need to discuss it in the results section (pg9). This could be potentially misleading.

o It is interesting that the regression results differed when the CAST or SDS was used. Please discuss your ideas on the cause of this difference.

Discussion:

o The discussion would benefit from more citations to support the points being made. Tying these findings to the published literature greatly helps understand the potential contribution of the paper.

o The aim was clearly stated in the discussion, providing good closure to the article. However, I would like more information on why it is important to investigate the ‘wide-ranging array’ of dysregulated behaviors and substance use at the same time (p9).

o “MultiCAGE score for illegal drugs was discarded due to its obvious overlap with use of cannabis” (pg 10). What was the correlation of these scales in your study? Additionally please remove the word ‘obvious’ from this sentence to improve adherence to academic style.

o For clarity, more discussion of why alcohol and nicotine were used as controls (pg10), preferably in the introduction or methodology would be useful.

o At the end of the first paragraph of limitations and strengths provide a citation for criticisms of the scales due to false positives (pg 13)

The current paper is interesting and answers a relevant topical question about the association between cannabis use and other potentially problematic behaviors. This research builds upon and contributes meaningfully to the literature. However, the paper requires significant revisions for clarity prior to publication.

Reviewer #2: This is a valuable study on a representative sample of University students in Spain, on the relationships between cannabis use and cannabis-related problems, with other potentially dysregulated behaviors (e.g., shopping, internet use, eating). The systematic analysis of gender is also a strength in this context.

Third line of 1st paragraph of Introduction: Please substitute the word “youngsters” with something more informative, e.g., adolescents, or persons in the age range X-Y etc.

Second paragraph of Introduction: Please introduce the fact that “gambling disorder” is in DSM5, as an example of how problematic behaviors can eventually be diagnosed. Also introduce the term “substance use disorders”, and not only “addiction”.

Please simplify or split this sentence in the last paragraph of the Introduction, page 3: “And some level of co-occurrence of problematic video gaming with substance use (although lower than the association between use of different substances between them and with gambling) also seems to exist, especially in young males.”

Please also simplify the next sentence in page 3: “For some authors [17-20], the interest in exploring these associations would be also justified by the potential psychological and neurobiological similarities between excessive involvement in these behaviors and well-established addictions.”

As a possible alternative for the above sentence: Some authors [17-20] have discussed the potential psychological and neurobiological similarities between excessive involvement in these behaviors and currently diagnosed addictions (“substance use disorders”).

“Participants” section in the Methods: Please mention if the questionnaires were administered by an assistant or if they were filled as pen-and paper by the participants themselves. Please explain if the level of privacy during these data collections.

First paragraph of page 4: When the authors mention “cannabis abuse and dependence”, are these meant to describe the DSM-IV diagnoses? Please state this clearly in text. Also, in the last sentence in the same paragraph, please briefly mention that the DSM5 and ICD “cannabis use disorder: is also now one diagnosed disorder, with increasing severity based on criteria. This is also relevant to the first sentence of the “Statistical Analysis” section in the Methods (please mention directly what scores were examined).

Methods: Could the authors provide a summary Table that shows the main instruments and their basic properties? For example, what they measure, score range, any cutoff values. This may be a helpful tool for the readers. Please also define any Abbreviations used (e.g., “MC” in the Table 2 and 3 legends).

Figure 1: Please provide further y-axis tick marks for the variables, and also summarize the statistical analysis of between-gender effects.

Discussion, page 11: Please simplify the following sentence, or split into different sentences: “A good candidate to play that role is weakened emotion regulation, and, more specifically, its manifestation as affect-driven impulsivity, namely the proneness to rash action when experiencing strong positive or negative emotions (positive and negative urgency).”

Page 12, first sentence in the section on “Gender”: Please change “we were unable

to detect” into “we did not detect”.

Please mention in the “Limitations” section if the fact that these were enrolled University Students could limit the generalization to non-University enrolled persons of the same age. For example, could non-University enrolled persons have more severe cannabis use disorders? (see a possible suggestion below).

Please also simplify and split this sentence on page 13 into different sentences, and substitute the term “overpathologising” with a more descriptive term: “Among limitations, , and second, that scales measuring putative behavioral addictions by transference of items from scales for substance use disorders (e.g. from CAGE-alcohol to MultiCAGE-video gaming or internet “addictions”) have been criticized for yielding a large number of false positives and overpathologising normal behavior.”

Just as a possible alternative: “We must also note that generalizability beyond the population of reference is not ensured (for example to non-University enrolled persons of the same age). Also, scales measuring putative behavioral addictions based on items developed for scales for substance use disorders (e.g. from CAGE-alcohol to MultiCAGE-video gaming or internet “addictions”) may yield a large number of “false positives” and increase the risk of pathologising normative behaviors.”

Reviewer #3: This study aimed to examine relations between addictive behaviors, cannabis abuse/dependence, and gender. The study has several strengths, including the large sample size and the statistical rigor; however, there are several other notable limitations and opportunities for increased explanation.

Introduction:

- At the end of paragraph 3, the authors comment about interests exploring psychological and neurobiological similarities between co-occurring addictive behaviors. I would like to see the authors expand a bit more on the potential variables that might account for these relations.

- The researchers appear interested in gender differences in associations between cannabis problems and other dysregulated behaviors. The introduction would benefit from data supporting why this would be an interesting analysis to conduct. Perhaps men and women use cannabis for different motives and thus, might experience different forms of other co-occurring dysregulated behaviors? Or is it related to their use patterns? Any research that might support looking at gender differences for this analysis would bolster the rationale for this analysis.

Method:

- How was the Fagerstrom Test for Nicotine Dependence scored/summed?

- I’m not sure I follow the scoring for the CAST. The authors describe the response scale as 1 to 5, with 1 indicating never. A cut-off score of 3 would indicate then that 3 “never” responses would indicate moderate cannabis addiction. Further explanation is necessary.

- What are the limitations of grouping individuals with scores of 2-4 on the Multicage CAD-4 in one group for analyses? While I understand the necessity to ensure adequate observations per group, categorizing potentially dissimilar levels of risk as the same might be problematic. If previous work supports this grouping, it should be cited in this paper.

Results:

- It would be helpful for the authors to provide the actual mean scores (either in the text or in the figure) for the CAST and SDS scores by gender, as they are a bit difficult to determine in the figure.

- Additionally, if I am understanding correctly, nearly no one in the sample would qualify as meeting even moderate cannabis abuse/dependence. If this is the case, the discussion might benefit from tempering of some language. For instance, “Cannabis abuse and dependence were more prevalent in males than females.” While it may be true that males outscored females on these measures, would it truly be the case that “abuse and dependence” were more prevalent or that males endorsed more symptoms of problematic cannabis use?

Discussion:

- The discussion could benefit from increased explanation for the greater association between cannabis and video gaming compared to alcohol and other externalizing problems. Is there any literature to support why this might be?

- Within the discussion of the potential transdiagnostic mechanism underlying addictions, are there any gender differences in emotion regulation/impulsivity that might also contribute to this study’s findings?

Minor Comments:

- In second paragraph about the MultiCAGE CAD-4, third sentence “additive disorders” should be “addictive.”

- In the fourth paragraph of the discussion, last sentence, the authors write, “In addition, the motives for Internet engagement seem to differ between males and females [52].” This sentence could benefit from elaboration on how those motives differ.

- In the first paragraph of “The role of gender” section, the authors write, “women in our study presented higher scores for eating disorders.” From the MultiCAGE CAD-4 description, it seems that this measure assesses risk of problematic eating behaviors and not eating disorders specifically. If this is the case, this language should be edited.

**********

6. PLOS authors have the option to publish the peer review history of their article (what does this mean?). If published, this will include your full peer review and any attached files.

If you choose “no”, your identity will remain anonymous but your review may still be made public.

**Do you want your identity to be public for this peer review?** For information about this choice, including consent withdrawal, please see our Privacy Policy.

Reviewer #1: No

Reviewer #2: No

Reviewer #3: No

[NOTE: If reviewer comments were submitted as an attachment file, they will be attached to this email and accessible via the submission site. Please log into your account, locate the manuscript record, and check for the action link "View Attachments". If this link does not appear, there are no attachment files.]

While revising your submission, please upload your figure files to the Preflight Analysis and Conversion Engine (PACE) digital diagnostic tool, https://pacev2.apexcovantage.com/. PACE helps ensure that figures meet PLOS requirements. To use PACE, you must first register as a user. Registration is free. Then, login and navigate to the UPLOAD tab, where you will find detailed instructions on how to use the tool. If you encounter any issues or have any questions when using PACE, please email PLOS at figures@plos.org. Please note that Supporting Information files do not need this step.

# Author comment: Author response to Decision Letter 0

**DOI:** 10.1371/journal.pone.0255872.r002

9 Jun 2021

On this screen, you may select previous files that you wish to keep. The next screen, which is accessed by clicking Proceed, will allow you to upload additional files.

If you previously submitted the manuscript in PDF, uncheck and replace the file in DOC, DOCX, or RTF on the next screen. This allows Editorial Manager to automatically fill in details under Manuscript Data.

If you revised a LaTeX manuscript, submit in PDF.

Attachment Submitted filename: Response to Reviewers.docx.doc Click here for additional data file.

# Peer review: Decision Letter 1

**DOI:** 10.1371/journal.pone.0255872.r003

**Authors:** Robert Didden

28 Jun 2021

PONE-D-20-32444R1

Association patterns of cannabis abuse and dependence with risk of problematic non-substancerelated dysregulated and addictive behaviors

PLOS ONE

Dear Dr. López-Torrecillas,

Thank you for submitting your manuscript to PLOS ONE. After careful consideration, we feel that it has merit but does not fully meet PLOS ONE’s publication criteria as it currently stands. Therefore, we invite you to submit a revised version of the manuscript that addresses the points raised during the review process.

When revising your manuscript, please check the manuscript for language errors and try to write concise where possible. And take a close look at Reviewer #1 comments on terminology, psychometrics, and decisions regarding some elements of the statistical analyses and data processing.

Please submit your revised manuscript by Aug 12 2021 11:59PM. If you will need more time than this to complete your revisions, please reply to this message or contact the journal office at plosone@plos.org. When you're ready to submit your revision, log on to https://www.editorialmanager.com/pone/ and select the 'Submissions Needing Revision' folder to locate your manuscript file.

Please include the following items when submitting your revised manuscript:

- A rebuttal letter that responds to each point raised by the academic editor and reviewer(s). You should upload this letter as a separate file labeled 'Response to Reviewers'.
- A marked-up copy of your manuscript that highlights changes made to the original version. You should upload this as a separate file labeled 'Revised Manuscript with Track Changes'.
- An unmarked version of your revised paper without tracked changes. You should upload this as a separate file labeled 'Manuscript'.

If you would like to make changes to your financial disclosure, please include your updated statement in your cover letter. Guidelines for resubmitting your figure files are available below the reviewer comments at the end of this letter.

If applicable, we recommend that you deposit your laboratory protocols in protocols.io to enhance the reproducibility of your results. Protocols.io assigns your protocol its own identifier (DOI) so that it can be cited independently in the future. For instructions see: http://journals.plos.org/plosone/s/submission-guidelines#loc-laboratory-protocols. Additionally, PLOS ONE offers an option for publishing peer-reviewed Lab Protocol articles, which describe protocols hosted on protocols.io. Read more information on sharing protocols at https://plos.org/protocols?utm_medium=editorial-email&utm_source=authorletters&utm_campaign=protocols.

We look forward to receiving your revised manuscript.

Kind regards,

Robert Didden

Academic Editor

PLOS ONE

Journal Requirements:

Please review your reference list to ensure that it is complete and correct. If you have cited papers that have been retracted, please include the rationale for doing so in the manuscript text, or remove these references and replace them with relevant current references. Any changes to the reference list should be mentioned in the rebuttal letter that accompanies your revised manuscript. If you need to cite a retracted article, indicate the article’s retracted status in the References list and also include a citation and full reference for the retraction notice.

[Note: HTML markup is below. Please do not edit.]

Reviewers' comments:

Reviewer's Responses to Questions

**Comments to the Author**

1. If the authors have adequately addressed your comments raised in a previous round of review and you feel that this manuscript is now acceptable for publication, you may indicate that here to bypass the “Comments to the Author” section, enter your conflict of interest statement in the “Confidential to Editor” section, and submit your "Accept" recommendation.

Reviewer #1: (No Response)

Reviewer #2: All comments have been addressed

Reviewer #3: All comments have been addressed

**********

2. Is the manuscript technically sound, and do the data support the conclusions?

The manuscript must describe a technically sound piece of scientific research with data that supports the conclusions. Experiments must have been conducted rigorously, with appropriate controls, replication, and sample sizes. The conclusions must be drawn appropriately based on the data presented. 

Reviewer #1: Yes

Reviewer #2: Yes

Reviewer #3: Yes

**********

3. Has the statistical analysis been performed appropriately and rigorously? 

Reviewer #1: Yes

Reviewer #2: Yes

Reviewer #3: Yes

**********

4. Have the authors made all data underlying the findings in their manuscript fully available?

The PLOS Data policy requires authors to make all data underlying the findings described in their manuscript fully available without restriction, with rare exception (please refer to the Data Availability Statement in the manuscript PDF file). The data should be provided as part of the manuscript or its supporting information, or deposited to a public repository. For example, in addition to summary statistics, the data points behind means, medians and variance measures should be available. If there are restrictions on publicly sharing data—e.g. participant privacy or use of data from a third party—those must be specified.

Reviewer #1: Yes

Reviewer #2: Yes

Reviewer #3: Yes

**********

5. Is the manuscript presented in an intelligible fashion and written in standard English?

PLOS ONE does not copyedit accepted manuscripts, so the language in submitted articles must be clear, correct, and unambiguous. Any typographical or grammatical errors should be corrected at revision, so please note any specific errors here.

Reviewer #1: Yes

Reviewer #2: Yes

Reviewer #3: Yes

**********

6. Review Comments to the Author

Please use the space provided to explain your answers to the questions above. You may also include additional comments for the author, including concerns about dual publication, research ethics, or publication ethics. (Please upload your review as an attachment if it exceeds 20,000 characters)

Reviewer #1: We appreciate the authors efforts in revising their manuscript and are satisfied with the changes made to address our prior comments. The authors did respond to the comments and suggestions presented in the previous review although the clarity of the paper remains marginal.

Areas for improvement of the current submission are as follows:

- Overall the paper’s readability and clarity needs improvement. We encourage the authors to re-read and review, particularly for excessively long sentences and any colloquial language.

- The first section of the introduction is much improved, however the paper would flow better if this section ended with a short sentence explaining why cannabis use was chosen as the main focus of the study.

- The ‘Present Study’ section could be significantly shortened. Detailed information about the measures used and their psychometrics would be better placed in the methods section, as having this much information in the introduction is distracting and confusing. Additionally, remove the details about the sample for the same reason.

- Page 6 “Actually, a privileged association….” Remove the word actually. This language is too colloquial for academic writing.

- Page 8 “Mean age of the sample was 21.12 years (DT= 7.23)” The letters DT appear to be erroneous, please correct.

- Page 8 rather than “non-assigned gender” use more neutral language such as: have a gender identity other than exclusively male or female

- Page 8 in the participants paragraph ‘patients’ and ‘participants’ are used interchangeably. Please change all to participants

- In the ‘Measures’ section we appreciate that the authors have gone to effort to include more psychometrics. However, reliability and validity are different concepts. For example for the multiCAGE CAD-4 the authors state that the measure has ‘good reliability and validity’ then only present internal reliability statistics. Please address both reliability and validity for each measure, with statistical values.

- Page 9 plasma cotinine levels correlations are used as a validity measure. It is my understanding that cotinine levels only indicate if someone has smoked recently (within a day or so) and is not useful for differentiating addiction. Please, briefly, note why this is a good measure for validation.

- Page 10: the sentence “Psychometric analyses……. Cannabis users” needs a citation at the end.

- Page 10: “given that our sample was extracted from the general population” the sample was a college sample not the general population, please clarify.

- In the statistical analysis section, it is unclear why the high scores were collapsed into a single level. The authors describe this as needed to ensure a sufficient number of observations per level, however if this results in the result being sorted into an invalid manner then it calls the interpretability of the results into question. Please justify this decision more fully and acknowledged the lack of pathology in the sample as a limitation of the study.

- Overall, the discussion is much improved, however as with the introduction readability could be further improved

- Across page 15/16 there is a sentence containing a quote from another paper speculating the personality of gamblers, please remove the quote, reference the paper and shorten this section to improve clarity.

- Page 16: the first sentence in “The role of gender” is unclear, please re-write and replace ‘intensity’ with strength.

- Page 17: “… theoretical interpretations beyond these general coincidences with previous research” change coincidences to similarities.

Reviewer #2: Page 8; Line 1: Please define the abbreviation “DT” (presumably a measure of variation).

Discussion, fourth line: “In addition, incidence of drug and gambling-related problems are sensitive to gender”. The authors could clarify this sentence, for example: “In addition, incidence of drug and gambling-related problems may differ by gender”.

Reviewer #3: The authors have adequately addressed all of my comments and concerns. I have no further comments at this time.

**********

7. PLOS authors have the option to publish the peer review history of their article (what does this mean?). If published, this will include your full peer review and any attached files.

If you choose “no”, your identity will remain anonymous but your review may still be made public.

**Do you want your identity to be public for this peer review?** For information about this choice, including consent withdrawal, please see our Privacy Policy.

Reviewer #1: No

Reviewer #2: No

Reviewer #3: No

[NOTE: If reviewer comments were submitted as an attachment file, they will be attached to this email and accessible via the submission site. Please log into your account, locate the manuscript record, and check for the action link "View Attachments". If this link does not appear, there are no attachment files.]

While revising your submission, please upload your figure files to the Preflight Analysis and Conversion Engine (PACE) digital diagnostic tool, https://pacev2.apexcovantage.com/. PACE helps ensure that figures meet PLOS requirements. To use PACE, you must first register as a user. Registration is free. Then, login and navigate to the UPLOAD tab, where you will find detailed instructions on how to use the tool. If you encounter any issues or have any questions when using PACE, please email PLOS at figures@plos.org. Please note that Supporting Information files do not need this step.

# Author comment: Author response to Decision Letter 1

**DOI:** 10.1371/journal.pone.0255872.r004

15 Jul 2021

Response to reviewers:

Reviewer 1

1) Overall the paper's readability and clarity needs improvement. We encourage the authors to re-read and review, particularly for excessively long sentences and any colloquial language.

We have substantially rewritten parts of the introduction and discussion sections to improve readability and flow. Changes are highlighted in yellow.

2) The first section of the introduction is much improved, however the paper would flow better if this section ended with a short sentence explaining why cannabis use was chosen as the main focus of the study.

We have added the following lines to the second-to-last paragraph of the background subsection:

“However, to date, research has tracked coincidences more closely than potential divergences between substance and non-substance related problems (and between different putative behavioral addictions). In this regard, previous studies have unveiled a distinctive link of problematic gaming and other sedentary leisure activities with cannabis use [3, 10]. This link, in turn, seems to be underpinned by personality and individual differences factors that can be dissociated from the ones responsible for a more general and well-known overlap between addictive behaviors [10]. The corroboration of this pattern of associations while controlling for relevant confounders is, however, still pending.”

3) The 'Present Study' section could be significantly shortened. Detailed information about the measures used and their psychometrics would be better placed in the methods section, as having this much information in the introduction is distracting and confusing. Additionally, remove the details about the sample for the same reason.

We have removed the information we have considered non-essential for the arguments made in the introduction.

4) Page 6 "Actually, a privileged association…." Remove the wordactually. This language is too colloquial for academic writing.

Done. 

5) Page 8 "Mean age of the sample was 21.12 years (DT= 7.23)" The letters DT appear to be erroneous, please correct.

 Done.

6) Page 8 rather than "non-assigned gender" use more neutral language such as: have a gender identity other than exclusively male or female.

Done.

7) Page 8 in the participants paragraph 'patients' and 'participants' are used interchangeably. Please change all to participants. 

Done.

8) In the 'Measures' section we appreciate that the authors have gone to effort to include more psychometrics. However, reliability and validity are different concepts. For example for the multiCAGE CAD-4 the authors state that the measure has 'good reliability and validity' then only present internal reliability statistics. Please address both reliability and validity for each measure, with statistical values.

We have added the required information, when available. See, for example, the rewritten part in the description of MultiCAGE: 

“To our knowledge, criterion validity has been established only for the substance use scales, not the behavioral scales. A cut-off score of 2 was observed to have 92.4% diagnostic sensitivity for alcohol use disorder, 100% for heroin and cannabis use disorder, and 94.1% for cocaine use disorder [34]. Among the non-substance related scales, Internet and video games related problems have been observed to weakly but significantly correlate with executive functioning, social behavior, and emotional control problems, as well as with general mental health [49].”

9) Page 9 plasma cotinine levels correlations are used as a validity measure. It is my understanding that cotinine levels only indicate if someone has smoked recently (within a day or so) and is not useful for differentiating addiction. Please, briefly, note why this is a good measure for validation.

The following sentence has been added to the corresponding section:

“Cotinine was used in this validation study instead of nicotine, as it is relatively insensitive to the immediate effects of smoking and constitutes a more stable measure of chronic intake [51].”

(Sentence extracted from the original reference).

10) Page 10: the sentence "Psychometric analyses……. Cannabis users" needs a citation at the end.

That sentence has been removed in the revised version.

11) Page 10: "given that our sample was extracted from the general population" the sample was a college sample not the general population, please clarify.

Corrected.

12) In the statistical analysis section, it is unclear why the high scores were collapsed into a single level. The authors describe this as needed to ensure a sufficient number of observations per level, however if this results in the result being sorted into an invalid manner then it calls the interpretability of the results into question. Please justify this decision more fully and acknowledged the lack of pathology in the sample

as a limitation of the study.

The following clarifications have been made in the corresponding section: 

“Please note that this analysis in particular could have been affected by a reduction of sensitivity of the scale to differences in the high end, so it is important it is confirmed by further analyses (in which all values were retained, and modelled using a Poisson distribution).”

In addition, a mention to the issue has been added to the limitations section.

13) Overall, the discussion is much improved, however as with the introduction readability could be further improved.

We have rewritten parts of this section in an attempt to improve flow and readability.

14) Across page 15/16 there is a sentence containing a quote from another paper speculating the personality of gamblers, please remove the quote, reference the paper and shorten this section to improve clarity.

That part has been rewritten as follows (the quote has been removed): 

“On the other hand, among multiCAGE dimensions, the video gaming score is the one least associated with alcohol-related and other externalizing problems. Yet, it is the only one clearly associated with cannabis abuse and dependence. A similar dissociation has been previously reported [3, 10], and strongly suggests diverging etiologies for different patterns of behavior customarily considered as addictive. More specifically, it seems unlikely for the association between cannabis and video gaming-related problems to be rooted in externalization and related traits (at difference with what seems to happen for the comorbidity between alcohol use disorder, gambling disorder, and conduct problems).”

15) Page 16: the first sentence in "The role of gender" is unclear, please re-write and replace 'intensity' with strength.

The first two sentences have been rewritten as follows:

“Gender differences were observed in all relevant measures (see Table 1). However, we did not detect significant differences between males and females regarding the strength of relationships between cannabis abuse/dependence and MultiCAGE scores.”

16) "… theoretical interpretations beyond these general coincidences with previous research" change coincidences to similarities.”

Corrected.

Reviewer 2

1) Discussion, fourth line: "In addition, incidence of drug and gambling-related problems are sensitive to gender". The authors could clarify this sentence, for example: "In addition, incidence of drug and gambling-related problems may differ by gender".

We have followed the reviewer’s advice at rewriting that sentence.

Reviewer 3

1) The authors have adequately addressed all of my comments and concerns. I have no further comments at this time.

Many thanks indeed for endorsing our article and for your constructive comments in the previous rounds.

Attachment Submitted filename: Response to reviewers PONE-D-20-32444.R2.doc Click here for additional data file.

# Peer review: Decision Letter 2

**DOI:** 10.1371/journal.pone.0255872.r005

**Authors:** Robert Didden

27 Jul 2021

Association patterns of cannabis abuse and dependence with risk of problematic non-substance-related dysregulated and addictive behaviors

PONE-D-20-32444R2

Dear Dr. López-Torrecillas,

We’re pleased to inform you that your manuscript has been judged scientifically suitable for publication and will be formally accepted for publication once it meets all outstanding technical requirements.

Within one week, you’ll receive an e-mail detailing the required amendments. When these have been addressed, you’ll receive a formal acceptance letter and your manuscript will be scheduled for publication.

An invoice for payment will follow shortly after the formal acceptance. To ensure an efficient process, please log into Editorial Manager at http://www.editorialmanager.com/pone/, click the 'Update My Information' link at the top of the page, and double check that your user information is up-to-date. If you have any billing related questions, please contact our Author Billing department directly at authorbilling@plos.org.

If your institution or institutions have a press office, please notify them about your upcoming paper to help maximize its impact. If they’ll be preparing press materials, please inform our press team as soon as possible -- no later than 48 hours after receiving the formal acceptance. Your manuscript will remain under strict press embargo until 2 pm Eastern Time on the date of publication. For more information, please contact onepress@plos.org.

Kind regards,

Robert Didden

Academic Editor

PLOS ONE

Additional Editor Comments (optional):

Reviewers' comments:

Reviewer's Responses to Questions

**Comments to the Author**

1. If the authors have adequately addressed your comments raised in a previous round of review and you feel that this manuscript is now acceptable for publication, you may indicate that here to bypass the “Comments to the Author” section, enter your conflict of interest statement in the “Confidential to Editor” section, and submit your "Accept" recommendation.

Reviewer #1: All comments have been addressed

Reviewer #2: All comments have been addressed

**********

2. Is the manuscript technically sound, and do the data support the conclusions?

The manuscript must describe a technically sound piece of scientific research with data that supports the conclusions. Experiments must have been conducted rigorously, with appropriate controls, replication, and sample sizes. The conclusions must be drawn appropriately based on the data presented. 

Reviewer #1: Yes

Reviewer #2: Yes

**********

3. Has the statistical analysis been performed appropriately and rigorously? 

Reviewer #1: Yes

Reviewer #2: Yes

**********

4. Have the authors made all data underlying the findings in their manuscript fully available?

The PLOS Data policy requires authors to make all data underlying the findings described in their manuscript fully available without restriction, with rare exception (please refer to the Data Availability Statement in the manuscript PDF file). The data should be provided as part of the manuscript or its supporting information, or deposited to a public repository. For example, in addition to summary statistics, the data points behind means, medians and variance measures should be available. If there are restrictions on publicly sharing data—e.g. participant privacy or use of data from a third party—those must be specified.

Reviewer #1: Yes

Reviewer #2: Yes

**********

5. Is the manuscript presented in an intelligible fashion and written in standard English?

PLOS ONE does not copyedit accepted manuscripts, so the language in submitted articles must be clear, correct, and unambiguous. Any typographical or grammatical errors should be corrected at revision, so please note any specific errors here.

Reviewer #1: Yes

Reviewer #2: No

**********

6. Review Comments to the Author

Please use the space provided to explain your answers to the questions above. You may also include additional comments for the author, including concerns about dual publication, research ethics, or publication ethics. (Please upload your review as an attachment if it exceeds 20,000 characters)

Reviewer #1: (No Response)

Reviewer #2: Some of the new sentences are not easily understood:

For example:

Page 4:

"However, to date, research has tracked coincidences more closely than potential divergences between substance

and non-substance related problems (and between different putative behavioral addictions). In this

regard, previous studies have unveiled a distinctive link of problematic gaming and other sedentary

leisure activities with cannabis use [3, 10]. This link, in turn, seems to be underpinned by personality

and individual differences factors that can be dissociated from the ones responsible for a more

general and well-known overlap between addictive behaviors [10]. The corroboration of these

associations while controlling for relevant confounders is, however, still pending."

Please simplify these sentences. In particular the first sentence needs editing (the term "coincidences" is not used commonly in this setting). Do the authors mean "associations"?

**********

7. PLOS authors have the option to publish the peer review history of their article (what does this mean?). If published, this will include your full peer review and any attached files.

If you choose “no”, your identity will remain anonymous but your review may still be made public.

**Do you want your identity to be public for this peer review?** For information about this choice, including consent withdrawal, please see our Privacy Policy.

Reviewer #1: No

Reviewer #2: No

# Editor report: Acceptance letter

**DOI:** 10.1371/journal.pone.0255872.r006

**Authors:** Robert Didden

30 Jul 2021

PONE-D-20-32444R2 

Association patterns of cannabis abuse and dependence with risk of problematic non-substance-related dysregulated and addictive behaviors 

Dear Dr. López-Torrecillas:

I'm pleased to inform you that your manuscript has been deemed suitable for publication in PLOS ONE. Congratulations! Your manuscript is now with our production department. 

If your institution or institutions have a press office, please let them know about your upcoming paper now to help maximize its impact. If they'll be preparing press materials, please inform our press team within the next 48 hours. Your manuscript will remain under strict press embargo until 2 pm Eastern Time on the date of publication. For more information please contact onepress@plos.org.

If we can help with anything else, please email us at plosone@plos.org. 

Thank you for submitting your work to PLOS ONE and supporting open access. 

Kind regards, 

PLOS ONE Editorial Office Staff

on behalf of

Professor Robert Didden 

Academic Editor

PLOS ONE
